# Temporal Dynamics of Recombination in Field Isolates of Foot-and-Mouth Disease Virus

**DOI:** 10.3390/v18020262

**Published:** 2026-02-19

**Authors:** Mate Malichava, Alexander Lukashev, Yulia Aleshina

**Affiliations:** 1 Martsinovsky Institute of Medical Parasitology, Tropical and Vector Borne Diseases, Sechenov First Moscow State Medical University, 119435 Moscow, Russia; malichava_m_m@student.sechenov.ru (M.M.);; 2 Research Institute for Systems Biology and Medicine, 117246 Moscow, Russia; 3 Faculty of Bioengineering and Bioinformatics, Lomonosov Moscow State University, 119234 Moscow, Russia

**Keywords:** FMDV, recombination, recombinant forms, emerging infections

## Abstract

Foot-and-mouth disease virus (FMDV) is a highly contagious pathogen of cloven-hoofed livestock. Recombination is one of the mechanisms that contribute to genetic diversity of FMDV and facilitate the generation of new viral lineages, or recombinant forms. While the general patterns of recombination in FMDV are well-known, the temporal dynamics of this process remain unexplored. This study systematically analyzed recombination across 1485 publicly available complete genome sequences of FMDV, collected from 1934 to 2024. In addition to the well-known general recombination pattern with hotspots on the borders of the genome region that encodes capsid proteins VP2-VP3-VP1, we identified serotype-specific recombination patterns. A significant temporal signal required to analyze temporal dynamics was found in serotypes A, Asia1, O, and SAT1 in the VP2-VP3-VP1 genome region. To assess the lifetimes of FMDV recombinant forms, we compared these time-scaled phylogenetic trees with phylogenies for other genomic regions exchanged by recombination events. The median lifetimes of FMDV recombinant forms ranged from 2 to 18 years, depending on the serotype and the nonstructural genomic region involved in recombination. These timescales are comparable to human (+)RNA viruses, such as enteroviruses and caliciviruses. In distinct serotypes, recombination could be more frequent on the 5′ or 3′ border of the capsid-encoding genome region, without a uniform pattern.

## 1. Introduction

Foot-and-mouth disease virus (FMDV) is a small non-enveloped virus with a single-stranded, positive-sense RNA genome that belongs to the species *Aphthovirus vesiculae*, family *Picornaviridae*. FMDV is a causative agent of Foot-and-mouth disease (FMD), a severe and contagious disease that affects cloven-hoofed animals, such as cattle, sheep, and pigs [1,2]. Although the disease is rarely fatal, it leads to substantial economic losses due to its detrimental effects on animal health, productivity, and international livestock trade [3]. FDMV is endemic in many countries of Africa, Asia, Middle East and some parts of South America. Europe, North America, and Oceania are recognized as FMD-free [4]. However, in 2025, two unrelated strains of FMDV serotype O caused isolated outbreaks in Germany, Slovakia, and Hungary, marking the first cases in decades for these countries [5,6,7].

The FMDV genome consists of a single-stranded RNA molecule approximately 8.4 kb in length, with VPg protein covalently attached to the 5′-end and a polyadenylated 3′-end. The genome contains a single large open reading frame (ORF) flanked by a structured 5′ untranslated region (UTRs) that contains an internal ribosomal binding site (IRES) and short 3′ UTR. ORF encodes a polyprotein that undergoes proteolytic cleavage by viral proteases to generate mature proteins and precursors. Based on the function of mature proteins, polyprotein can be divided into four regions (L, P1, P2, and P3). The P1 region encodes the structural proteins VP1–VP4, which assemble into the viral capsid and are critical for host cell receptor binding and immune recognition [8,9]. The L, P2, and P3 regions encode nonstructural proteins involved in polyprotein processing (mediated by 2Apro, 3Cpro, 3CDpro) and genome replication (2B, 2C, 3AB, 3B/VPg, 3CDpro, and 3Dpol) [10].

Based on antigenic properties, FMDV is classified into seven serotypes, namely O, A, C, Asia1 (Euro-Asiatic serotypes), Southern African Territories (SAT) 1, SAT2, and SAT3 (Southern African serotypes), with no cross protection between different serotypes of FMDV [11]. Serotypes of FMDV can be distinguished based on nucleotide sequence variation in the VP1 region that contains major antigenic epitopes [8,12,13,14,15] and is therefore the primary target for molecular epidemiology studies. FMDV serotypes exhibit distinct global distributions: serotypes O and A occur worldwide (except southern Africa), Asia1 is confined to Asia, while SAT1-2 circulate throughout Africa and SAT3 remains restricted to eastern/southern regions of Africa [4]. Serotype C is considered to be extinct and has not been detected since 2004 [16]. Serotypes are further divided into topotypes and lineages based on geography and VP1 coding sequence [17].

Homologous recombination is a key evolutionary mechanism in FMDV, contributing to its genetic diversity and adaptation. Although this process occurs predominantly between viruses of the same serotype [18], inter-serotypic recombinants have been identified among field isolates [19,20,21,22,23,24,25,26] and reproduced in experimental settings [27,28,29,30,31]. Moreover, recombination between globally spread (A, O, Asia1) and SAT serotypes occurs more rarely than within these groups [18]. The recombination patterns in FMDV are well-known and resemble those of other picornaviruses, with recombination hotspots located at the boundaries of genomic regions encoding structural and nonstructural proteins. While recombination occurs freely in nonstructural regions—albeit without prominent hotspots—it is almost absent within structural regions. Consequently, structural and nonstructural proteins have distinct evolutionary histories, and the genome segments encoding structural proteins are suggested to function as interchangeable modules [18,22,32].

Recombination provides an important mechanism for the emergence of novel virus variants with better fitness, facilitating their establishment and circulation within a host population [21,26,33]. For instance, intra-serotypic recombination may have led to the emergence of Iran-05 lineage (serotype A) [34], which was first identified in Iran in 2003 and subsequently spread throughout the Middle East and South Asia [35,36]. Furthermore, viruses belonging to serotype O PanAsia-2 lineage responsible for outbreaks in West Eurasia and introduced into FMD-free Bulgaria in 2011 were found to have a chimeric genome with nonstructural regions acquired from viruses of serotypes Asia1 and A [25,37].

The complex evolutionary history of FMDV genomes presents significant challenges for developing effective vaccines and controlling the disease. Moreover, the frequency at which recombination generates novel established circulating lineages—termed recombinant forms—remains unknown. Understanding the temporal dynamics of this process is therefore crucial for epidemiology. In this study, we analyze recombination across all publicly available FMDV complete genome sequences, focusing on differences in recombination patterns between serotypes. Furthermore, we implemented an automated method to assess the temporal dynamics of recombination. This method is based upon a published manual approach [38,39] which was developed here to automatically compare time-scaled phylogenetic trees from recombinant genomic regions to calculate the lifetimes and half-life of recombinant forms. We show that the half-lives of FMDV recombinant forms range from 2 to 18 years, depending on the serotype and the nonstructural genomic region involved in recombination.

## 2. Materials and Methods

### 2.1. Data Collection

#### 2.1.1. Alignment of Coding Sequences

All publicly available complete genome sequences of FMDV (*n* = 1870) with exclusion of synthetic constructs were downloaded from the GenBank database (accessed on 5 March 2024) using the following search term: ((“Foot-and-mouth disease virus”[Organism] OR FMDV[All Fields]) AND biomol_genomic[PROP] AND (“6900”[SLEN]: “11000”[SLEN])) NOT (“synthetic construct”[Organism] OR artificial[All Fields] OR patent[All Fields] OR unverified[All Fields]). Corresponding metadata was also collected from GenBank entries and manually examined (Table S1). Sequences were additionally filtered by “isolation source” qualifier and “TITLE” field in GenBank entry to exclude those from inactivated vaccines, experimentally infected animals, and laboratory-derived strains.

Nucleotide coding sequences (CDS) were excised from the complete genomes based on coordinates in the Genbank entry annotations and manually curated. These CDS were then translated, and the resulting amino acid sequences were aligned using MAFFT v7.520 [40]. Finally, the protein alignment was reverse translated to nucleotides using a custom Python 3 script (https://github.com/v-julia/alignment_of_orfs/blob/master/trans_alignment.py (accessed on 22 December 2025)). Sequences with more than five ambiguous nucleotides or more than two ambiguous nucleotides in a row were removed from the dataset (62 sequences). The remaining ambiguous nucleotides in 330 sequences were resolved according to the closest sequence within a 100 nt window surrounding the ambiguous nucleotide (Python 3 script is available at https://github.com/v-julia/resolve_ambiguous (accessed on 22 December 2025)). Sequences with long deletions (more than 50 gaps in a row) (*n* = 50) were also omitted from the analysis. A total of 132 out of 164 missing sampling dates of isolates were manually retrieved from the literature. Since sampling dates are essential for the reconstruction of time-scaled phylogenies, sequences with missing sampling dates were omitted. The resulting alignment of ORFs encoding the polyprotein comprised 1439 sequences and was examined for quality manually in JalView v.2.11.5.1 and BioEdit v.7.2.5 programs. The geographic pools of viruses in the dataset were determined according to [41].

A significant number of virus isolates (*n* = 261) originated from probang-derived samples. These samples most likely represent persistent infections, as this method is routinely used to screen for potential FMDV carriers. Since the FMDV carriers are not likely to take part in natural transmission, the persistent infection can last for years and could affect the molecular dating analyses [42]. These sequences were excluded from molecular dating analysis. Also, sequences associated with the FMD outbreak in the UK in August 2007 caused by a release of vaccine strain O1 BFS [43] were excluded prior to recombination temporal dynamics analysis.

#### 2.1.2. Inference of Missing FMDV Typing Data

Since serotype designation was missing for 32.7% of sequences, and topotype and lineage data are usually not indicated in GenBank entry annotation, we implemented a semi-automatic annotation pipeline. First, VP1 genomic regions of representative strains/isolates for each recognized FMDV topotype (as curated by FMDV Nomenclature Working Group and available at [44]) were retrieved from GenBank (pandas, requests, bs4, entrez python packages). These VP1 sequences (147) were then incorporated into the multiple sequence alignment of VP1 sequence excised from our ORF alignment. A maximum-likelihood phylogenetic tree was built from this VP1 alignment (see Section 2.3 for details). Using FigTree v1.4.4 [45], we manually assigned serotype and topotype labels to clades based on their clustering with the representative isolates. Finally, these annotations were extracted from the phylogeny (in NEXUS format) and mapped to the corresponding sequence metadata table. The pipeline is available at https://github.com/Finkonn/FMDV_recombination (accessed on 22 December 2025).

#### 2.1.3. Subsampling of Serotype O Sequences for Bayesian Phylogenetic Analysis

Since the dataset of sequences for serotype O (*n* = 777) was large and required excessive time for Bayesian tree inference, the phylogenetic analysis and recombinant forms’ lifetimes estimation was performed on four downsampled datasets of 98 sequences. Two downsampling strategies were utilized: random selection of 50 sequences and reduction of sequences from overrepresented studies. For the latter, sequences were grouped by the first five characters in their GenBank accession codes. All sequences from groups smaller than the threshold (two sequences) were retained. For each of the larger studies, the reduced dataset was supplemented by either one sequence or 10% of randomly chosen sequences from that study. While both strategies produced datasets with a sufficient temporal signal, a study-based approach was shown to produce more consistent estimates of evolutionary parameters [46]. Consequently, the datasets generated by this method were used for further Bayesian phylogenetic inference.

Sequence alignments and scripts for data preparation are available at https://github.com/Finkonn/FMDV_recombination (accessed on 22 December 2025).

### 2.2. Recombination Analysis

For species-level recombination analysis, similar sequences with identity higher than 97% were deleted from the alignment of ORFs, resulting in an alignment of 325 sequences. A full exploratory recombination analysis was performed on this alignment using the RDP4 v.101 software [47] with nine standard recombination detection methods: RDP [48], GENECONV [49], MaxChi [50], Chimaera [51], BootScan [52], SiScan [53], 3seq [54], PhylPro [55], and LARD [54]. Default parameters were used, with sequences specified as linear. Only events supported by at least five methods were considered significant.

For serotype-level analysis, the corresponding ORF alignments were extracted from the full dataset (*n* = 1439 sequences with known collection dates). The same procedure was then repeated independently for each serotype-specific alignment. The breakpoint distribution was manually inspected to identify recombination-free regions. The alignment was subsequently split at the boundaries of these regions.

### 2.3. Phylogenetic Analysis

Maximum likelihood (ML) phylogenies for the VP1 nucleotide sequence and for recombination-free regions identified from species-level and serotype-level breakpoint plots were inferred using IQ-TREE v3.0.1 [56]. The best-fit substitution models were selected automatically under the Bayesian Information Criterion (BIC) using the ModelFinder tool [57] integrated in IQ-TREE, and branch support was assessed by an ultrafast bootstrap approximation with 1000 replicates [58].

### 2.4. Molecular Dating

Prior to Bayesian phylogenetic analysis, the presence of a temporal signal in each alignment of recombination-free region was assessed using TempEst v.1.5.3 [59]. For each dataset, the optimal root was selected using the “correlation” function, and the corresponding root-to-tip distances were exported. The regression of root-to-tip distance against sampling time was repeated using the ordinary least squares algorithm (scikit-learn Python package) and visualized (matplotlib Python package). For each dataset, Spearman’s rank correlation coefficient, regression slope, and the associated *p*-values were calculated. A dataset was considered to have a sufficient temporal signal if a *p*-value for the regression slope was lower than 0.05. Examination of serotype-level alignments revealed several outliers with anomalous branch lengths given their collection date. All such outliers were removed prior to Bayesian phylogenetic analysis. For the four random subsamples of serotype O, this reduced their size from an initial 98 sequences each to final datasets of 82, 76, 80, and 79 sequences.

For each serotype-level alignment of the recombination-free part of the P1 region (VP2–VP1–VP3), partitioning schemes and substitution models were evaluated using PartitionFinder 2 [60] with the greedy search algorithm, and the best-fit configuration (partition: 1,2,3; substitution model: GTR + G + X) was selected according to the corrected Akaike Information Criterion (AICc).

Time-scaled phylogenies were inferred using BEAST v2.7.7 [61]. To select the optimal evolutionary model, log marginal likelihoods across all combinations of molecular clock models (strict, relaxed lognormal, relaxed exponential) and coalescent tree priors (constant size, exponential growth, Bayesian skyline) were estimated for each alignment using Nested Sampling implementation in BEAST2 [62]. The final combination of molecular clock model and tree prior for each alignment was chosen based on the Bayes factor test (Table S2).

Subsequently, the temporal signal was validated for each dataset using Bayesian Evaluation of Temporal Signal (BETS) [63,64] under the selected best-fit model. Briefly, we compared marginal likelihoods of the best molecular clock model under two configurations of sampling dates: the correct sampling dates and no sampling dates (Table S2). The dataset was considered to have temporal signal if log Bayes factor (>5) supports a model with correct sampling dates.

For each alignment with temporal signal confirmed using BETS, independent Markov chain Monte Carlo (MCMC) chains were run. The parameters of MCMC runs differed among serotypes and are summarized in Table S2. MCMC convergence was assessed in Tracer v1.7.2 [65], confirming that the effective sample size of all parameters was at least 200. Maximum clade credibility (MCC) trees were annotated with TreeAnnotator v.2.7.7. using 10% burn-in.

Phylogenetic trees were visualized using FigTree v1.4.4 software [45], treeio v.1.28.0 [66] and ggtree v.3.12.0 [67] R (version 4.4.1) packages.

### 2.5. Estimation of Recombinant Forms Half-Lives

Recombination leads to the generation of novel recombinant viruses with chimeric genomes. A novel chimeric virus that is viable may be fixed by founder effect or natural selection and subsequently spread through a population of susceptible hosts. Virus lineages that arise from recombination between distinct and recognizable parental genomes are referred to as recombinant forms. The temporal dynamics of virus recombination can be characterized by the recombinant forms’ half-life. Generally, two approaches for calculating the half-lives of recombinant forms of small RNA viruses are presented in the literature and have been applied to several serotypes of enteroviruses and noroviruses [38,39,68,69,70]. The first approach is based on calculating pairwise genetic distances in the genomic region encoding VP1 capsid protein. This genome region is frequently used in surveillance; it almost never has recombination within it and has sufficient temporal signal for inferring a time-scaled phylogeny. The proportion of recombinant viruses at different pairwise distances in VP1 is then calculated. Viruses are considered recombinant if they have different genotypes based on capsid and polymerase, which are defined manually by experts. Then, the recombinant form half-life is calculated as a ratio of VP1 distance corresponding to 50% of recombinant viruses, which is divided by two, because both non-recombinant descendants diverged from their common ancestor.

**Figure 1:**
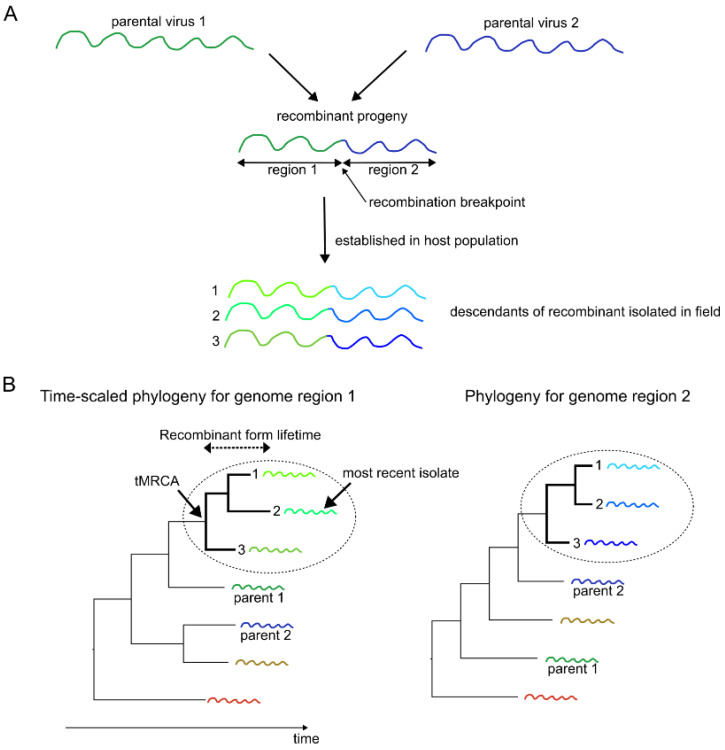
Schematic representation of recombinant form detection and their lifetime estimation. (**A**) In this example, a recombinant virus inherited the 5′ end of its genome from one parent (parental virus 1) and the 3′ end from the other (parental virus 2). If established in a population, its descendants can be identified through field surveillance and sequencing. (**B**) Since the inherited genomic regions have distinct evolutionary histories, the position of a recombinant sequences on a phylogenetic tree depends on the genome region being analyzed: in a tree built using the 5′ region (region 1), descendants cluster with parental virus 1, whereas in a tree built using the 3′ region (region 2), they group with parental virus 2. The resulting lineage forms a clade with consistent branching in both phylogenetic trees (trees for each exchanged/inherited regions) and is referred to as a recombinant form. Using the time-scaled phylogeny for the region with sufficient temporal signal (region 1), recombinant form lifetime is estimated as the difference between the time to most common recent ancestor (tMRCA) of the clade and the sampling date of the most recent isolate of the lineage.

The second approach identifies recombinant forms by comparing the phylogenetic trees built for two genomic regions that were likely exchanged by recombination and determining the clades that maintained consistent branching patterns (Figure 1) [38,39]. The lifetime of a recombinant form—the duration of its circulation in the host population—is calculated as the time between the most recent common ancestor (tMRCA) of the clade (time of the recombinant form emergence) and the date of its most recent isolate. The calculation of the lifetimes of recombinant forms requires a time-scaled phylogeny which is typically built for a genomic region with a sufficient temporal signal, such as VP1. The half-life of recombinant forms is then estimated as a median lifetime of all observed recombinant forms. The advantage of this approach is that it does not require designation of virus genotypes/genogroups/lineages, which typically requires expert knowledge and may be arbitrary for FMDV, especially in the genome regions other than VP1 because only VP1 is routinely used for classification.

In the original studies [38,39], tree comparisons were performed manually, which is unsuitable for analyzing large sequence datasets. Here, we present RF-HL, a tool for identifying common subtrees in two phylogenetic trees and calculating the half-lives of recombinant forms. RF-HL requires two input phylogenetic trees: one in Newick format (which can be inferred using any algorithm) and a second, time-scaled phylogeny in Nexus format inferred using BEAST. It provides two methods for identifying recombinant forms:

1. Search for common subtrees: This method identifies common subtrees with consistent branching patterns. Each node is represented by its associated taxon splits; the splits from branches with weak statistical support are filtered out.
2. Search for common bipartitions: This method identifies shared bipartitions (the division of taxa into two sets defined by a branch). Common bipartitions corresponding to nested nodes are removed. This approach requires additional manual verification, as matching bipartitions can occur near the root and may not represent recent recombination events.

The program outputs a text file with common subtrees in Newick format and text file with recombinant forms’ lifetimes. The coinciding subtrees can be further visualized in the R environment. The source code with examples is available at https://github.com/v-julia/RF_HL (accessed on 15 February 2026).

## 3. Results

### 3.1. Data and FMDV Subtyping

The patterns of recombination in FMDV are well-studied, and the most recent comprehensive analysis of recombination was performed on the accumulated 84-year worth (1934–2017) genomic data of FMDV [18]. Since 2017, the number of complete genome sequences of FMDV (excluding vaccine and experimental strains) deposited in the NCBI Nucleotide database has almost doubled, reaching 1870 sequences (accessed as of March of 2024). After quality control and omitting genomes with missing sampling dates, the resulting dataset comprised 1439 sequences. For almost a third of the sequences, the serotype was not indicated in the annotation (Table 1). Since information on FMDV topotypes and lineages is also crucial for a more detailed analysis of recombination dynamics, we implemented a semi-automated pipeline for FMDV typing. Briefly, the serotypes, topotypes, and lineages were determined based on grouping with the representative strains curated by FMDV Nomenclature Working Group in the phylogenetic tree for the VP1 genomic region [44]. Serotype O was the most represented (54%), with 446 out of 802 sequences belonging to the Middle East–South Asia (ME-SA) topotype. This topotype includes O/ME-SA/PanAsia, O/ME-SA/Ind-2001, and O/ME-SA/PanAsia-2 lineages, which were responsible for the most significant FMD outbreaks over the last 25 years and thus were more extensively sequenced. The next most prevalent serotype was A (19.5%), featuring the Iran-05 lineage that was responsible for numerous outbreaks in the many countries of Middle East and South Asia in the 2000s (Table S1).

### 3.2. Recombination Analysis

**Figure 2:**
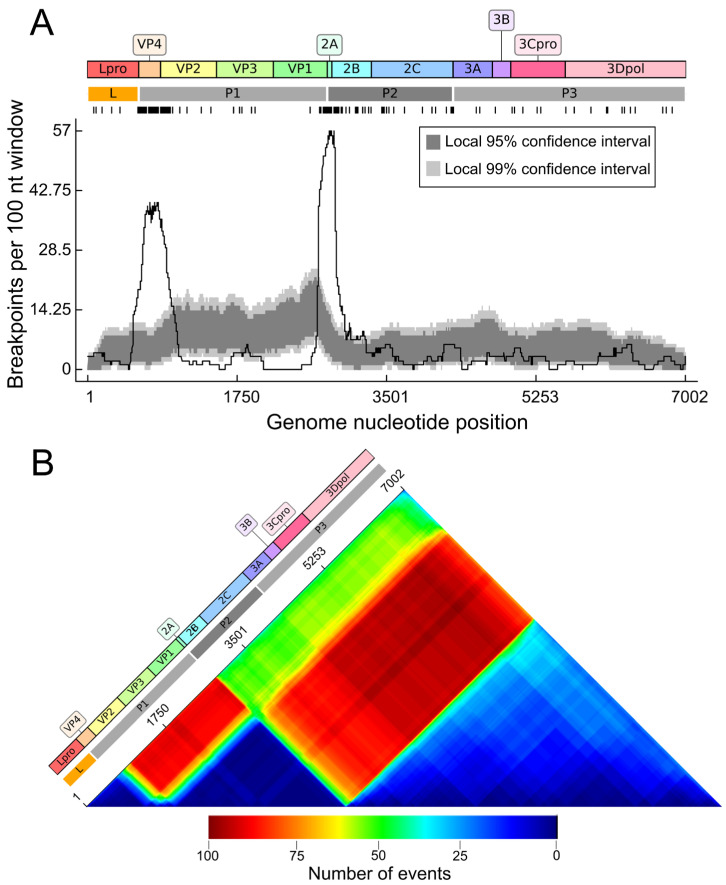
Recombination analysis of FMDV coding sequences using RDP4. (**A**) Distribution of inferred recombination breakpoints across the FMDV open reading frame (ORF). The top panel is a schematic of the FMDV open reading frame (ORF), showing the major functional regions (L, P1, P2, and P3) and mature proteins. Individual breakpoint locations are marked by tick marks below the plot. The bottom panel plots the number of inferred recombination breakpoints (black line) detected within a sliding 100 nt window. The light and dark gray areas represent the local 95% and 99% confidence intervals, respectively. Peaks where the black line rises above these shaded areas indicate statistically significant recombination hotspots; dips below indicate coldspots [47]. (**B**) Recombinant regions count matrix. Unique recombination events are mapped according to the inferred breakpoints at panel A. Each cell in the matrix corresponds to a pair of genome sites. The color of a cell (see heat scale) indicates the number of times the detected recombination events separated that specific pair of genome positions. Warmer colors (red) indicate genome regions that were more often exchanged due to recombination. The FMDV ORF schematic is aligned above for genomic reference.

The exploratory recombination analysis on the updated dataset showed similar recombination patterns as observed in earlier studies [18,22]. A total of 140 recombination events were supported by five or more methods. The most prominent recombination hotspots were detected in the VP4/5′ end of VP2 (nucleotide positions 575–975 in alignment) and in the 2A region (nucleotide positions 2743–2947) (Figure 2A). Conversely, most of the P1 region except VP4 was a significant recombination coldspot, with only eight breakpoints found in VP3. Similarly, breakpoint densities within Lpro, P2, and P3 were low, falling near the lower confidence intervals in permutation tests [47], confirming them as species-level coldspots (Figure 2A).

Analysis of the recombination region count matrix, which shows the number of times specific nucleotide pairs are separated by recombination events, indicated that the VP2-VP3-VP1 region was the most frequently transferred segment (Figure 2B). It was commonly separated from the Lpro region, the P2 region (excluding 2A), and the P3 regions, with a somewhat higher frequency of transfers relative to the P3 region. Transfers of the Lpro region relative to P2 and P3 were also detected, though at a lower frequency. Thus, the identified recombination hotspots divide the FMDV ORF into three blocks that correspond to its functional regions: (1) the L region encoding the leader protease Lpro, (2) the P1 region (VP2–VP3–VP1, excluding VP4) encoding the structural proteins, and (3) the P2–P3 region (excluding 2A) encoding the nonstructural proteins. The recombination analysis set up the three genome regions that were further used to analyze the temporal dynamics of recombination.

**Figure 3:**
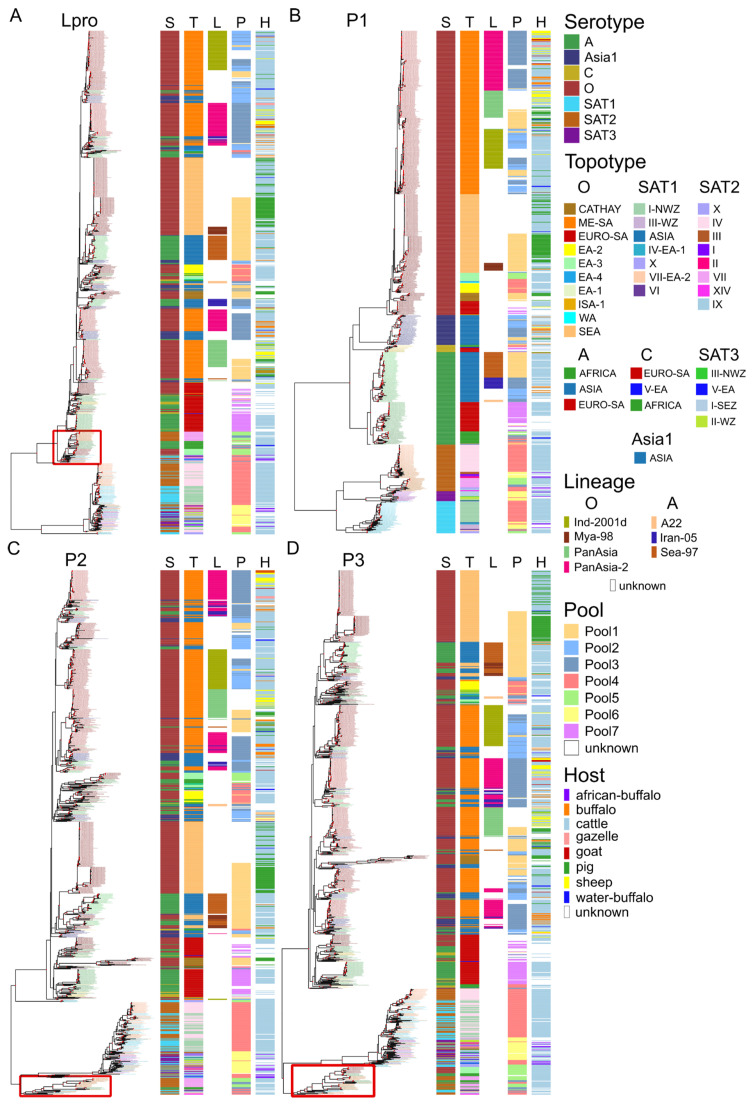
Maximum likelihood phylogenetic trees of FMDVs with available complete genomes. Four phylogenies were built using IQ-TREE v.3.0.1 for recombination-free alignments of the following genome regions: Lpro (**A**), P1 (except VP4) (**B**), P2 (**C**), and P3 (**D**). Tip labels are colored according to serotype (colors) and order of tree tips (shades) in the P1 phylogenetic tree. Serotypes (S), topotypes (T), particular lineages (L), geographic origin (P), and host (H) of sequences are visualized as color bars. Geographic regions are defined as follows: Pool1, East Asia; Pool2, South Asia; Pool3, West Eurasia; Pool4, East Africa; Pool5, West Africa; Pool6, Southern Africa; Pool7, South America [41]. Clades that comprise viruses of both globally spread and SAT serotypes are marked with red rectangles. Nodes with high ultrafast bootstrap support (>95%) are marked with red circles.

To characterize the extent of inter- and intra-serotypic recombination in the generation of recombinant forms, we constructed ML phylogenetic trees based on genomic regions with almost no recombination (Figure 3 and Figure S1). Even though there was no significant recombination hotspot between P2 and P3, they were analyzed separately due to the presence of several breakpoints on their boundary (Figure 2) and to be consistent with previous studies [18].

The phylogenetic tree of the P1 region revealed a well-supported segregation of seven FMDV serotypes, clearly separating globally spread serotypes (A, Asia1, O, and C) and SAT serotypes (Figure 3B and Figure S1). FMDV circulation has known geographic patterns, which may be described as endemic pools (viruses of different serotypes that circulate in the same region) and topotypes (VP1 lineages within a serotype that occupy the same geographic niche). The phylogeny of P1 perfectly reflected the topotype and lineage classification (Figure 3, bars T and L). However, the capsid-based topotype does not always correspond to a geographic endemic pool, for example, because viruses commonly spread beyond the region of origin that gave name to a topotype (Figure 3, bar P).

In contrast to P1, the trees for Lpro, P2, and P3 regions showed intermingling of different serotypes, forming at least three well-supported clades (ultrafast bootstrap support = 100). Two major clades consisted of global serotypes and of SAT viruses. Within these clades, viruses of different serotypes were intermixed. This pattern suggests transfers of the P1 region relative to the rest of genome between either global serotypes or SAT serotypes but not between global and SAT, consistent with previous studies [18].

In addition to these two major clades, there were additional clades in Lpro, P2, and P3 regions that bore signs of multiple recombination events involving global and SAT serotypes (Figure 3, red rectangles; Figure S2). In the Lpro region, it was a reliably supported group (ultrafast bootstrap support > 99) that included viruses from serotypes O, A, SAT1, SAT2, and SAT3 (Figure S2A). These viruses were isolated between 1960 and 2022 in East Africa (Kenya, Uganda, Ethiopia), Central Africa (Niger), and Middle East (Iraq, Israel). The grouping of viruses within this clade suggested several recombination events between global and SAT serotypes. In the P2 region, there was a third clade that included SAT1 and SAT2 sequences and several serotype A sequences from Ethiopia and Egypt (isolated in 2011–2019), compatible with a single recombination event between SAT and global clade viruses that were co-circulating in North and West Africa (Figure S2B). The P3 region showed a group of serotype A and O isolates from Ethiopia, Niger, and Algeria that were intermingled with SAT2 viruses from the same countries, suggesting about five recombination events (Figure S2C). Collectively, these results confirm that while recombination between global and African viruses is limited by geographic segregation, it is not restricted and has occurred multiple times.

There was no consistent connection between recombination and geographic groups among global serotypes. Some endemic pools and topotypes had evidence of multiple recombination events (Figure 3, bars P and T). For example, topotype ME-SA of serotype O, which had MRCA dating back 65–67 years, showed evidence of at least six recombination events with other topotypes indicating that its capsid was associated with various nonstructural genomic variants. An opposite example was topotype EURO-SA, designated in three serotypes (O, A, and C), which has monophyletic L, P2 and P3 genome regions (except for a few isolated recombination events in single sequences). 

### 3.3. Recombination Patterns Differ Between Serotypes

The overall analysis of FMDV recombination confirmed the known frequent nonstructural protein exchange between viruses of different serotypes. However, isolated recombination events that were distinct from the universal pattern could be overlooked in the whole FMDV dataset. Therefore, we conducted the analysis of recombination on separate datasets of FMDV sequences of distinct serotypes.

**Figure 4:**
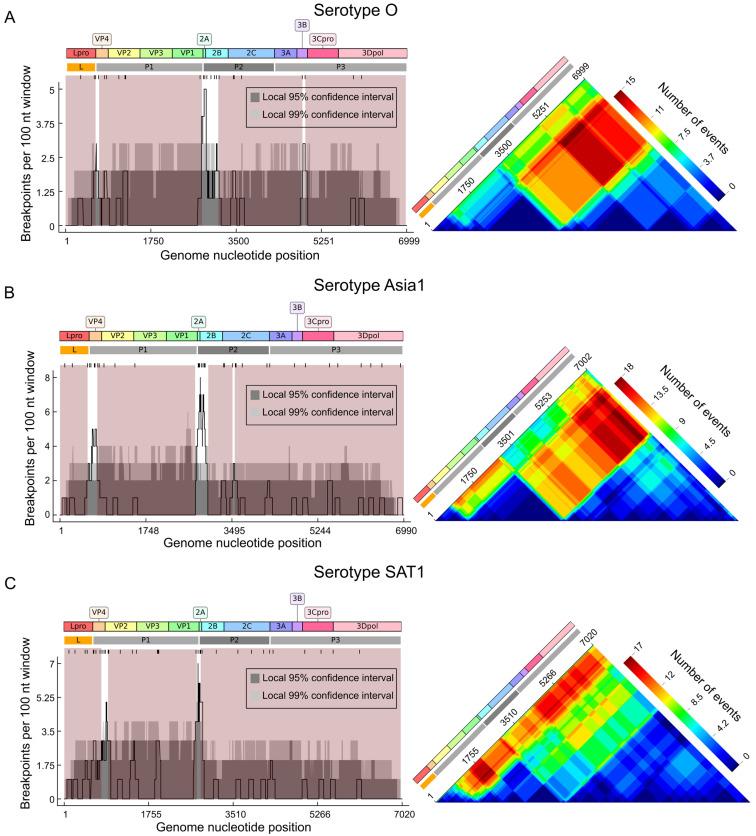
Distribution of recombination breakpoints and heatmap of recombination events detected by RDP4 in the coding sequences of FMDVs that belong to serotype O (**A**), Asia1 (**B**), and SAT1 (**C**). Left panel—Distribution of inferred recombination breakpoints across FMDV open reading frame (ORF). At the top is a schematic of the FMDV open reading frame (ORF), showing the major functional regions (L, P1, P2 and P3) and mature proteins. Individual breakpoint locations are marked by tick marks below the schematic. At the bottom is a plot of the number of inferred recombination breakpoints (black line) detected withing a sliding 100 nt window. The light and dark gray areas represent the local 95% and 99% confidence intervals, respectively. Peaks where the black line rises above these areas indicate statistically significant recombination hotspots; dips below indicate coldspots [47]. The genome regions with minimal recombination are shaded red. Right panel—Recombinant region count matrix. Unique recombination events are mapped according to the inferred breakpoints. Each cell in the matrix corresponds to a pair of genome sites. The color of a cell (see heat scale) indicates the number of times the detected recombination events separated that specific pair of positions. Warmer colors (red) indicate genome regions that are more frequently exchanged due to recombination. The FMDV ORF schematic is aligned above for genomic reference.

First, it was necessary to more accurately identify the recombination-free genome regions within the datasets representing distinct serotypes. While the general patterns of recombination—specifically the hotspots in VP4 and 2A—were conserved across serotypes, a more detailed analysis revealed additional hotspots in serotype-specific datasets (Figure 4). Particularly, the distribution of recombination points within L, P1, P2, and P3 blocks and the number of block exchanges differed. Beyond the conserved universal hotspots, significant breakpoint clustering was observed in the 3B region in the dataset of serotype O sequences and at the 2C/3A junction for the dataset of SAT2 sequences, where the breakpoint density exceeded the 95% confidence interval for random recombination corrected for sequence similarity (Figure 4A). There was also a concentration of breakpoints at the 2C/3A junction and in the 3Dpol observed for the serotype A dataset, and in 3Cpro of serotype SAT2, but the breakpoint density did not exceed the 95% confidence interval (Figure S3). These results allowed us to more accurately identify the boundaries of genomic regions with minimal recombination (Figure 4 and Figure S3, highlighted in red) as candidates for molecular dating and further calculation of the recombinant forms’ lifetimes. Importantly, recombination region count matrices indicated no evidence that Lpro was consistently linked with P2-P3 genome regions (i.e., they were less recombinant relative to each other than to P1), justifying their independent analysis. Due to limited sequence availability (SAT3, *n* = 22; C, *n* = 17), the recombination signals for these serotypes were insufficient for identification of reliable pattern, and they were consequently excluded from the subsequent temporal dynamics analysis.

### 3.4. Inference of Time-Scaled Phylogenies for FMDV P1 Regions

To identify genomic regions suitable for molecular dating, the temporal signals of Lpro, VP2–VP3–VP1(P1), P2, and P3 were assessed using the regression analysis of virus collection dates and root-to-tip distances in the ML phylogenetic trees. Notably, the initial dataset comprised 261 isolates obtained from probang samples, which most possibly represent persistently infected animals. Since the persistent FMDV infection can last up to several years [42] and it is not clear how to date such isolates, these isolates were excluded from molecular dating analysis. All genome regions demonstrated a statistically significant temporal signal (Figure S4, *p*-values of regression slopes and Pearson correlation <0.05). The inferred substitution rates were: 1.55 × 10^−3^ substitutions per site per year (s/s/y) for Lpro, 1.91 × 10^−3^ s/s/y for P1, 1.15 × 10^−3^ s/s/y for P2, and 7.1 × 10^−4^ s/s/y for P3. These rates are consistent with previous studies, which reported 1.71 × 10^−3^ s/s/y for P1 and ~3–4 × 10^−4^ s/s/y for the P3 region [18]. However, our results found a significant temporal signal in the Lpro and P2 regions, which were previously reported to lack one.

**Figure 5:**
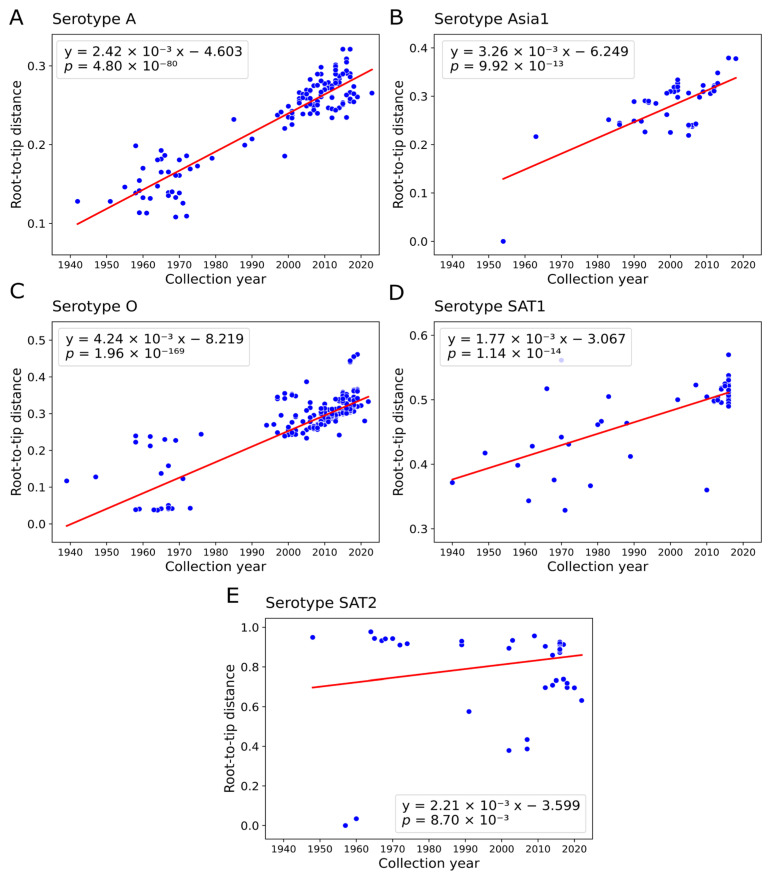
Evaluation of temporal signal in VP2-VP3-VP1(P1) region of FMDV serotypes A (**A**), Asia1 (**B**), O (**C**), SAT1 (**D**), SAT2 (**E**). Root-to-tip regression analysis of collection dates and root-to-tip divergences in ML phylogenetic trees was performed in TempEst v.1.5.3 software [59]. The red line represents the linear regression fit, with its equation and statistical significance (*p*-value) shown in each plot. The slope of regression indicates the substitution rate (s/s/y).

The temporal signal was present in P1 in the whole dataset, but it had to be confirmed in datasets of distinct serotype sequences. In most serotype datasets there was a reliable correlation between root-to-tip distances and sampling dates upon TempEst analysis. However, the correlation was weak (though significant) in SAT2 (Pearson ρ = 0.22, *p*-value = 0.009) (Figure 5E). To formally evaluate temporal signal, we performed a Bayesian Estimation of Temporal Signal (BETS) analysis implemented in BEAST2, which compares a model that utilizes the true sampling dates to a null model where all dates are set equal. This test confirmed a detectable temporal signal for all serotypes, except for serotype SAT2 (Table S2). Consequently, SAT2 was excluded from subsequent analysis of recombination temporal dynamics. For a large dataset of O serotype sequences (*n* = 802), computational constraints prevented Bayesian phylogenetic analysis. To address this, we performed the analysis on four randomly downsampled datasets (76–82 sequences each), confirming that the temporal signal was robust to the downsampling.

### 3.5. Dating the Lifetimes of FMDV Recombinant Forms

**Figure 6:**
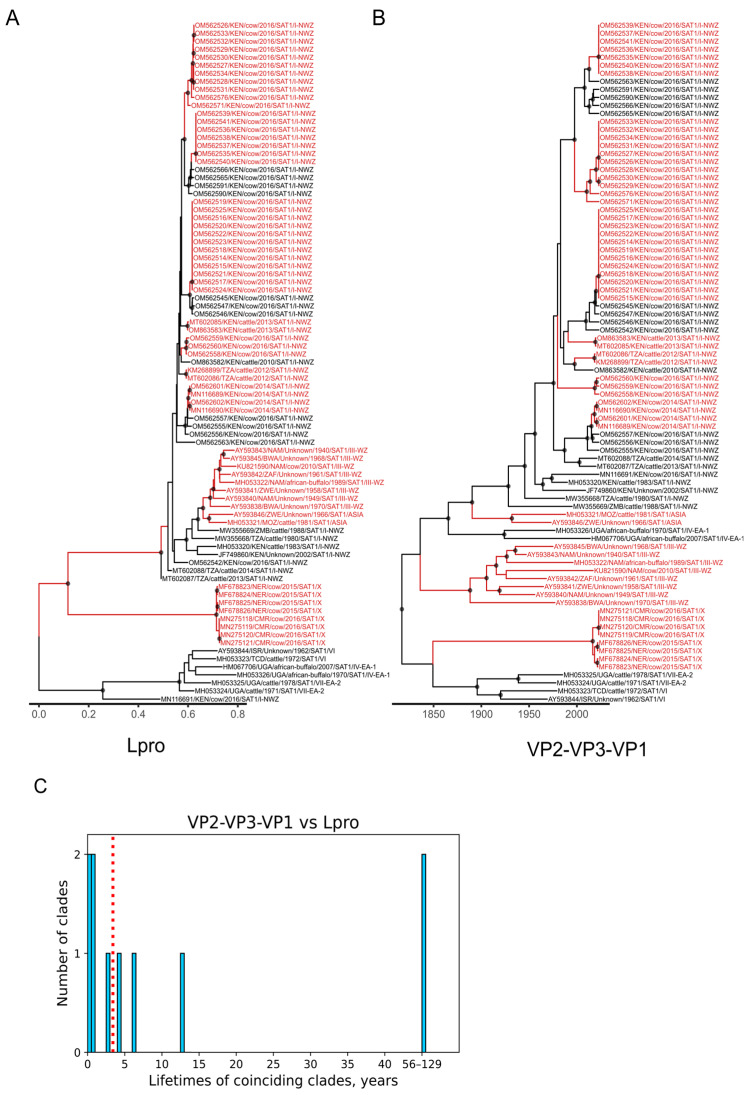
Inference of half-life of recombinant forms that occurred due to recombination between Lpro and the structural genomic region (VP2-VP3-VP1) in serotype SAT1 FMDV sequences. (**A**) ML tree built using Lpro genome region. (**B**) Bayesian time-scaled phylogeny inferred using the VP2-VP3-VP1 region. In both trees, black circles indicate nodes with high support (ultrafast bootstrap > 95% in A, posterior probability > 0.9 in B). Clades (bipartitions) that coincide between trees are colored red. (**C**) Distribution of lifetimes (time between tMRCA and most recent isolate) for clades that coincide between the Lpro and VP2-VP3-VP1. Recombinant forms’ half-life (median lifetime of clades) is shown as a dashed red line.

Time-scaled phylogenies for the P1 (VP2-VP3-VP1) region yielded substitution rates typical to FMDV P1 (Table 2) and allowed calculation of the lifetimes of FMDV recombinant forms (circulating virus lineages that originated from a recombination event and did not undergo additional recombination events). We tested several approaches for comparing trees. First, we searched for the subtrees with identical topology. While straightforward, this method often identified numerous small clades of only 2–3 sequences and proved error-prone, as it did not account for phylogenetic uncertainty, especially in the case of large clades that included many similar sequences with poorly resolved phylogenetic relations and low bootstrap supports. A conservative interpretation of the topology (relying strictly on well-supported groups) in this case would split a large clade into many small subclades. However, the biologically meaningful recombinant form is likely the entire larger clade, and the apparent topological differences that lack bootstrap support are more likely to be artifacts of insufficient phylogenetic signal, rather than evidence of further recombination. To address this limitation, we adopted a bipartition-based approach. We treated each tree as a set of branches (bipartitions), each splitting the leaves into two groups. We then searched specifically for bipartitions that coincided between trees and were strongly supported (ultrafast bootstrap > 95% or posterior probability > 0.9) (Figure 6A,B).

In FMDV, the recombinant forms typically emerged through recombination at the junction between the Lpro, VP2-VP3-VP1, P2 (without 2A), and P3 genomic regions, with additional contributions from the recombination at the P2/P3 junction in datasets for serotypes A and SAT2, and a recombination breakpoint in the 3D RdRp-coding region in datasets for serotype A. Accordingly, we identified coinciding clades by comparing the P1 time-scaled phylogenies with ML trees constructed for the other genomic regions (Figure 6A,B and Figures S6–S12). We then defined the lifetime of a recombinant form as the time between its most recent isolate and the most recent common ancestor (tMRCA) of the recombinant form. Recombinant forms’ half-lives were subsequently calculated as the median of these lifetimes across all identified RFs (Table 3, Figure 6C and Figure S5).

In general, the lifetimes of coinciding clades ranged from several months to 129 years (Figure 6C and Figure S5). The recombinant forms’ half-lives (median lifetimes of recombinant forms) ranged from 1.7 years to 18 years, indicating that recombination often generated short-lived lineages (Table 3). The half-lives of recombinant forms varied considerably by serotype and genome region involved in recombination. Serotypes Asia1 and A showed the shortest recombinant forms’ half-lives ranging from 1.7 to 5.54 years. In serotype A, the half-lives were similar across recombinant forms arising from exchanges involving different nonstructural regions. In Asia1, recombinant forms arising from Lpro/P1 recombination had a markedly longer half-life (5.83 years)—approximately 3.5 times that of recombinant forms originating from P2-P3/P1 exchange. Conversely, in serotypes O and SAT1, recombinant forms generated through Lpro/P1 recombination exhibited shorter median lifetimes than those involving other nonstructural junctions. To assess the effect of sampling bias, we performed half-life estimation analysis for four subsampled datasets of serotype O sequences. The absolute estimates varied across subsamples, with standard deviations of 0.79–1.59 years and a maximum 1.5-fold difference between replicates for the same genome region. The overall pattern—shorter recombinant forms’ half-lives in Lpro and longer in 3D—was consistent. It is noteworthy that in serotype O, the half-lives of recombinant forms were almost two times longer (thus, the recombination was less common) between P1 and 3C–3D compared to P1 and 2C–3A, although there were two recombination hotspots between P1 and 3C–3D and just one recombination hotspot between P1 and 2C–3A. Overall, sample bias could indeed affect the observed recombinant forms’ lifetimes; therefore, precise numbers should be interpreted with care, but their overall level remained highly reproducible across serotypes and genome regions.

## 4. Discussion

Recombination is a well-known feature of (+)RNA viruses, including FMDV, driving their evolution and genetic diversity [32,71]. Recombination in FMDV has been demonstrated both in field [19,20,21,22,23,24,25,26] and experimental studies [27,28,29,30,31]. Consistent with patterns observed in other picornaviruses, in FMDV, there are two well-defined recombination hotspots at the junctions of structural and nonstructural genomic regions, dividing FMDV genome into three functional blocks: the 5′NTR-Lpro region (most studies use the coding genome part and thus just Lpro), the P1 capsid region (VP2–VP3–VP1), and the P2–P3 nonstructural region [18,22]. Our analysis revealed notable variations in recombination hotspots for serotype-specific datasets. Beyond the mentioned hotspots, significant clustering of recombination breakpoints was identified at specific loci, such as within the 3B region in serotype O and at the 2C/3A junction in serotypes A and SAT2. Although these hotspots passed a formal significance test, they were made up by just a few recombination events; therefore, it may be premature to draw further conclusions from their detection. Furthermore, the frequency of genomic block exchanges varied among serotypes. For instance, while serotypes O and A exhibited more frequent exchanges of the P1 block relative to the P3 rather than Lpro region, Asia1 and SAT2 showed a comparable frequency of P1 exchange with both Lpro and P3. In SAT1, the Lpro region was the most commonly transferred. Collectively, the breakpoints at both edges of the P1 region appear comparably important, but the recombination landscape in FMDV is more complex. As the sequence sampling was notably biased for some serotypes, we can only conclude that there is no consistent difference between recombination frequency on the 5′ and 3′ boundary of the P1 genome region.

Our study corroborated the previous findings of predominant global genetic pools of FMDV nonstructural genes—one containing the globally disseminated serotypes (A, Asia1, and O) and the other containing the SAT serotypes—within which most recombination occurs [18,71]. The reproductive isolation between these pools has been largely attributed to geographic isolation with additional early experimental evidence indicating that recombination between more distantly related serotypes (O and SAT1) occurs at a lower frequency [31]. However, phylogenetic analysis of nonstructural genomic regions (Lpro, P2, and P3) identified at least one additional genetic cluster in each nonstructural region that comprised intermingled viruses with capsids from both global (O and A) serotypes isolated in Africa and African SAT serotypes, indicating recombination events between SAT and globally spread serotypes. A prominent example was observed in the P3 region, where sequences from serotypes A and O (isolated from Ethiopia, Niger, Egypt, and Algeria) clustered with SAT2 viruses from the same geographic regions, suggesting at least five independent recombination events (Figure S2C). These results suggest that although recombination between global and SAT lineages is limited, there is no strict barrier, and such recombination has occurred on multiple occasions between viruses co-circulating in one area.

Evidence of inter-serotype recombination was previously shown for panzootic lineages of FMDV, particularly for PanAsia2/O and Iran05/A [25,34,37]. The systematic analysis of FMDV genetic sequences showed that intra- and inter-serotypic recombination events occurred multiple times in the evolution of these lineages (Figure 3), with their isolates intermixed with other isolates of globally spread serotypes A, Asia1, and O. While the panzootic lineage PanAsia is conserved all over the genome, another major panzootic lineage PanAsia2 is a holistic lineage only in P1 and bears evidence of numerous recombination events in the nonstructural genome regions.

The goal of this study was to assess the temporal dynamics of FMDV recombination by estimating lifetimes of recombinant forms. The dynamics of recombination was extensively studied in several serotypes of enteroviruses and noroviruses [38,39,68,69,70]. These viruses are characterized by a prominent recombination hotspot at the junction between structural and nonstructural genomic regions, which enables classification into recombinant forms based on capsid and polymerase genes. Given the more complex recombination patterns in FMDV, we adopted a more general approach that defines recombinant forms as coinciding clades in phylogenetic trees built using exchanged genomic regions [38,39].

We dated the MRCA of recombinant forms using a time-scaled phylogeny of the structural genome region. The estimates of tMRCA of distinct serotypes were presented across several studies based on Bayesian inference using the VP1 coding sequence (Table 4). While our tMRCA estimates do not match precisely the earlier studies, their respective confidence intervals generally overlap, and the estimates of substitution rates were more consistent with published studies. The recombinant forms typically represent more recent clades and show narrower tMRCA intervals; therefore, recombinant forms’ lifetimes should be less prone to errors. Although prior studies have estimated rates for SAT2 and SAT3, we were unable to detect a temporal signal for these serotypes in our dataset. This limitation likely stems from our use of exclusively complete genome sequences, which resulted in a reduced dataset size compared to other studies that incorporated partial genomic data.

The lifetime of a recombinant form was estimated as the time interval between the tMRCA of a corresponding clade and the collection date of the most recent isolate within the recombinant form. It is crucial to note that these estimates represent a lower boundary because a recombinant form likely existed prior to the diversification of the sampled viruses (MRCA) that define its clade and could have been circulating after isolation of its most recent representative. Consequently, the true persistence of a lineage may be longer than our estimates indicate. On the other hand, due to poorly resolved phylogeny in some clades, we adopted a soft definition of recombinant forms, which could have additional recombination events within them. If such events involved closely related viruses, they could remain undetected. Another limitation could come from a separate analysis of serotypes. This was done to avoid data with poor temporal signal (SAT2 and SAT3) and additional breakpoints but could exclude inter-serotype recombination events. However, inter-serotype recombination would likely be detected anyway because such viruses would form distinct clades in the corresponding genome regions. Overall, the methodology was relatively robust as the standard deviations of recombinant forms’ half-lives ranged from 0.79 to 1.58 years between the O serotypes subsampled datasets, with the overall pattern (shorter half-lives for Lpro recombinants, longer for 3D recombinants) remaining consistent. This indicates that while absolute estimates carry some uncertainty due to sampling, the comparative conclusions are reliable.

A critical factor for estimation of recombination forms’ lifetimes is the quality of the time-scaled phylogeny itself. Sequences of non-natural origin (e.g., laboratory strains or potential lab leaks), incorrect collection dates, or viruses from persistently infected hosts can distort molecular clock estimates and bias half-life calculations. To mitigate this, we excluded sequences from potential FMDV carriers from the molecular dating analysis. This filtering improved the temporal signal and Markov chain Monte Carlo (MCMC) convergence. For serotype Asia1, where a significant proportion of sequences was removed (53 of 122), we observed a substantial decrease in estimated median recombinant form lifetimes. For serotype O, filtering improved consistency of the results by reducing the variance between subsamples. These results underscore the importance of dataset curation for molecular clock inference.

The half-life time of recombinant forms for serotype A was more than twice as short as for serotype O. While this could be a result of sampling bias, it is interesting to note that host diversity was higher for serotype O. A total of 86% of serotype A isolates and just 66% of serotype O isolates with a known host originated from cattle, while 1% of serotype A and 20% of serotype O isolates came from pigs. Also, serotype O, but not A, full-genome sequences originated from sheep 5% and goats (1%). It is possible that circulation in diverse hosts limited probability of recombination and provided longer circulation of distinct recombinant forms; however, given the highly uneven sampling coverage in terms of countries, hosts and outbreaks, this observation requires further confirmation.

The range of median lifetimes of FMDV recombinant forms is remarkably consistent with the evolutionary dynamics observed in enteroviruses and noroviruses, despite ecological differences and distinct recombination patterns. Comparative data show that enterovirus recombinant form half-lives span from ~1 year (echovirus 9) and 3–5 years (echovirus 30) to ~10 years (echovirus 11 and EV-A71 genotype C) [68,69,70]. Norovirus recombinant forms exhibit slightly longer half-lives of 8–10 years (GI/GII genogroups) [39]. This places FMDV recombinant turnover (2–18 years) within the same order of magnitude as these human pathogens.

## Figures and Tables

**Table 1:** Summary of FMDV complete genomic data available in the NCBI Nucleotide database.

Serotype	Number of Sequences with Explicitly Indicated Type (Percentage of Total)	Number of Sequences After Automated Typing (Percentage of Total)
O	611 (42.46)	777 (54)
A	173 (12.02)	278 (19.31)
C	14 (0.99)	16 (1.11)
Asia1	22 (1.53)	119 (8.27)
SAT1	31 (2.15)	88 (6.12)
SAT2	33 (2.29)	142 (9.87)
SAT3	19 (1.32)	19 (1.32)
Unknown	536 (37.24)	0 (0)

**Table 2:** Substitution rates and tMRCA inferred for recombination-free structural region (Figure 4) of FMDV serotypes, estimated using Bayesian phylogenetic inference (BEAST2). For serotype O, results are shown for analyses performed on four randomly subsampled datasets.

Serotype	Substitution Rate [95% Hpd Confidence Interval] × 10^−3^, s/s/y	tMRCA [95% HPD Confidence Interval], Years
O	2.76 [2.1–3.41]	117 [92.6–145.3]
	3.22 [2.54–3.94]	107 [88.36–127.99]
	3.37 [2.65–4.1]	98 [81.95–117.23]
	2.83 [2.22–3.52]	113 [91.26–138.4]
A	4.35 [2.7–6.46]	139 [102.87–186.6]
Asia1	3.60 [2.49–4.78]	104 [73.99–143.99]
SAT1	1.78 [1.01–2.65]	206 [130.5–295.53]

**Table 3:** The median lifetimes of recombinant forms resulting from recombination of nonstructural genome regions relative to the structural region in serotypes with sufficient temporal signal.

Serotype	Nonstructural Genome Region	Median Half-Life Time of Recombinant Forms, Years
Asia1	Lpro	5.83
	3′ part of 2C (P2)–P3	1.71
A	Lpro	5.54
	2C	4.07
	3A–3B–3Cpro–5′ part of 3D (P3)	3.97
	3′ part of 3D	3.97
O	Lpro	9.67
		8.39
		6.92
		6.08
		Mean = 7.77, SD =1.58
	2C (P2)–3A (P3)	11.08
		12.23
		9.57
		10.38
		Mean = 10.81, SD = 1.13
	3C–3D (P3)	16.17
		16.62
		17.98
		16.57
		Mean = 16.83, SD = 0.79
SAT1	Lpro	3.40
	2C (P2)–P3	11.31

**Table 4:** Published and current evolutionary estimates for FMDV serotypes, including the time to the most recent common ancestor (tMRCA) and evolutionary substitution rates (s/s/y). For Bayesian estimates, 95% HPD intervals are indicated for both tMRCA and substitution rates.

Serotype	Genomic Region	tMRCA [95% Confidence Interval], Years Before Present (ybp)	Substitution Rate [95% Confidence Interval] × 10^−3^, s/s/y	Method	Study
O	3′VP4-VP2-VP3-VP1	109 [88–132]	3.05 [2.37–3.74]	Bayesian inference	Present study
	VP2-VP3-VP1	132 [35–240]	Not applicable *	Maximum Likelihood	[18]
	VP1	92 [72–173]	3.14 [1.84–4.34]	Bayesian inference	[72]
	VP1	Not applicable **	3.69	Bayesian inference	[73]
SAT1	VP2-VP3-VP1	206 [131–296]	1.78 [1.01–2.65]	Bayesian inference	Present study
	VP2-VP3-VP1	388 [276–533]	Not applicable *	Maximum Likelihood	[18]
	VP1	538 [228–897]	1.30 [0.54–2.18]	Bayesian inference	[74]
	VP1	263 [185–363]	1.8 [1.52–2.99]	Bayesian inference	[73]
	VP1	141 [73–255]	6.50 [2.03–2.84]	Bayesian inference	[72]
Asia1	VP2-VP3-VP1	104 [74–144]	3.6 [2.49–4.78]	Bayesian inference	Present study
	VP2-VP3-VP1	84 [(-13)–188]	Not applicable *	Maximum Likelihood	[18]
	VP1	96 [54–161]	6.32 [3.39–9.47]	Bayesian inference	[72]
A	VP2-VP3-VP1	139 [103–187]	4.35 [2.7–6.46]	Bayesian inference	Present study
	VP2-VP3-VP1	232 [350–131]	Not applicable *	Maximum Likelihood	[18]
	VP1	178 [78–189]	4.26 [2.46–6.16]	Bayesian inference	[72]
	VP1	Not applicable **	4.67 [4.63–4.7]	Bayesian inference	[73]
A, O, Asia1		Not applicable ***	6.52 [4.85–8.40]	Bayesian inference	[75]

* In this study, the substitution rate was inferred for the whole FMDV dataset and was 1.71 × 10^−3^ s/s/y. ** Isolates of serotype O circulating in Africa were analyzed in this study. *** Isolates of serotypes O, A, and Asia1 circulating in Southeast Asia were considered in this study.

## Data Availability

The original data presented in the study are openly available at https://github.com/Finkonn/FMDV_recombination (accessed on 22 December 2025).

## Supplementary Materials

The following supporting information can be downloaded at: https://www.mdpi.com/article/10.3390/v18020262/s1, Table S1: FMDV sequences used in analysis metadata; Table S2: Model selection for BEAST analysis; Figure S1: Maximum likelihood phylogenetic trees of FMDVs with available complete genomes. Four phylogenies were built using IQ-TREE v.3.0.1 for recombination-free alignments of the following genome regions: Lpro (A), P1 (except VP4) (B), P2 (C), and P3 (D). Nodes with high ultrafast bootstrap support (>95%) are marked with black circles; Figure S2: Magnified clades that comprise globally spread serotypes (A, O, Asia1) and SAT serotypes in phylogenetic trees for Lpro (A), P2 (B), and P3 (C) genome regions. Nodes with high ultrafast bootstrap support (>95%) are marked with black circles; Figure S3: Distribution of recombination breakpoints and heatmap of recombination events detected by RDP4 in the coding sequences of FMDVs that belong to serotype A (A) and SAT2 (B). Left panel—Distribution of inferred recombination breakpoints across FMDV open reading frame (ORF). At the top is a schematic of the FMDV open reading frame (ORF), showing the major functional regions (L, P1, P2 and P3) and mature proteins. Individual breakpoint locations are marked by tick marks below the schematic. At the bottom is a plot of the number of inferred recombination breakpoints (black line) detected withing a sliding 100 nt window. The light and dark gray areas represent the local 95% and 99% confidence intervals, respectively. Peaks where the black line rises above these areas indicate statistically significant recombination hotspots; dips below indicate coldspots. The genome regions with minimal recombination are shaded red. Right panel—Recombinant region count matrix. Unique recombination events are mapped according to the inferred breakpoints. Each cell in the matrix corresponds to a pair of genome positions. The color of a cell (see heat scale) indicates the number of times the detected recombination events separated that specific pair of positions. Warmer colors (red) indicate genome regions that are more frequently exchanged due to recombination. The FMDV ORF schematic is aligned above for genomic reference; Figure S4: Evaluation of temporal signal in FMDV genomic regions with minimal recombination. Root-to-tip regression analyses were performed on maximum likelihood phylogenetic trees constructed for the (A) Lpro, (B) P1, (C) P2, and (D) P3 genomic regions. Each plot displays the linear regression equation and the statistical significance (*p*-value) of the slope. The slope of regression indicates the substitution rate (s/s/y); Figure S5: Distribution of lifetimes of coinciding clades (recombinant forms) in phylogenetic trees built for structural genome regions (VP2–VP3–VP1) and nonstructural regions with minimal recombination events in serotypes A (A), Asia1 (B), four downsampled datasets for serotype O (C–F) and SAT1 (G). The genomic regions involved in comparison are indicated on the top of each histogram. Recombinant form half-life (median lifetime of clade) is shown as a dashed red line; Figure S6: Comparison of time-scaled Bayesian phylogeny inferred using structural genomic region (VP2–VP3–VP1) (left) and ML tree (right) inferred using nonstructural genomic regions Lpro (A) and 3′ part of 2C (P2)–P3 (B) for serotype Asia1. In both trees, black circles indicate nodes with high support (ultrafast bootstrap > 95% or posterior probability > 0.9). Clades that coincide between trees are colored red; Figure S7: Comparison of time-scaled Bayesian phylogeny inferred using structural genomic region (VP2-VP3-VP1) (left) and ML tree (right) inferred using nonstructural genomic regions Lpro (A), 2C (B), 3A–5′ part of 3D (C), and 3′ part of 3D (D) for serotype A. In both trees, black circles indicate nodes with high support (ultrafast bootstrap > 95% or posterior probability > 0.9). Clades that coincide between trees are colored red. Virus lineages are visualized as color bars; Figure S8: Comparison of time-scaled Bayesian phylogeny inferred using structural genomic region (VP2–VP3–VP1) (left) and ML tree (right) inferred using nonstructural genomic regions Lpro (A), 2C–3A (B), and 3C-3D (C) for downsampled dataset of serotype O sequences (replicate 1). In both trees, black circles indicate nodes with high support (ultrafast bootstrap > 95% or posterior probability > 0.9). Clades that coincide between trees are colored red. Virus lineages are visualized as color bars; Figure S9: Comparison of time-scaled Bayesian phylogeny inferred using structural genomic region (VP2-VP3-VP1) (left) and ML tree (right) inferred using nonstructural genomic regions Lpro (A), 2C-3A (B), and 3C-3D (C) for downsampled dataset of serotype O sequences (replicate 2). In both trees, black circles indicate nodes with high support (ultrafast bootstrap > 95% or posterior probability > 0.9). Clades that coincide between trees are colored red. Virus lineages are visualized as color bars; Figure S10: Comparison of time-scaled Bayesian phylogeny inferred using structural genomic region (VP2–VP3–VP1) (left) and ML tree (right) inferred using nonstructural genomic regions Lpro (A), 2C-3A (B), and 3C–3D (C) for downsampled dataset of serotype O sequences (replicate 3). In both trees, black circles indicate nodes with high support (ultrafast bootstrap > 95% or posterior probability > 0.9). Clades that coincide between trees are colored red. Virus lineages are visualized as color bars; Figure S11: Comparison of time-scaled Bayesian phylogeny inferred using structural genomic region (VP2–VP3–VP1) (left) and ML tree (right) inferred using nonstructural genomic regions Lpro (A), 2C–3A (B), and 3C–3D (C) for downsampled dataset of serotype O sequences (replicate 4). In both trees, black circles indicate nodes with high support (ultrafast bootstrap > 95% or posterior probability > 0.9). Clades that coincide between trees are colored red. Virus lineages are visualized as color bars; Figure S12: Comparison of time-scaled Bayesian phylogeny inferred using structural genomic region (VP2–VP3–VP1) (left) and ML tree (right) inferred using nonstructural genomic regions Lpro (A) and 2C–P3 (B) for serotype SAT1. In both trees, black circles indicate nodes with high support (ultrafast bootstrap > 95% or posterior probability > 0.9). Clades that coincide between trees are colored red.

## Abbreviations

The following abbreviations are used in this manuscript:

FMDV	Foot-and-mouth disease virus
FMD	Foot-and-mouth disease
VPg	Viral protein genome-linked
ORF	Open reading frame
UTR	Untranslated region
SAT	Southern African Territories
CDS	Coding sequence
ML	Maximum likelihood
BIC	Bayesian information criterion
AICc	Corrected Akaike information criterion
BETS	Bayesian evaluation of temporal signal
MCMC	Markov chain Monte-Carlo
MCC	Maximum clade credibility
MRCA	Most recent common ancestor
tMRCA	Time to the most recent common ancestor
