# Supplementary figures and images for: Temporal Dynamics of Recombination in Field Isolates of Foot-and-Mouth Disease Virus
Source: Viruses. 2026 Feb 19;18(2):262. doi: 10.3390/v18020262 (PMC12945239; doi:10.3390/v18020262)

A

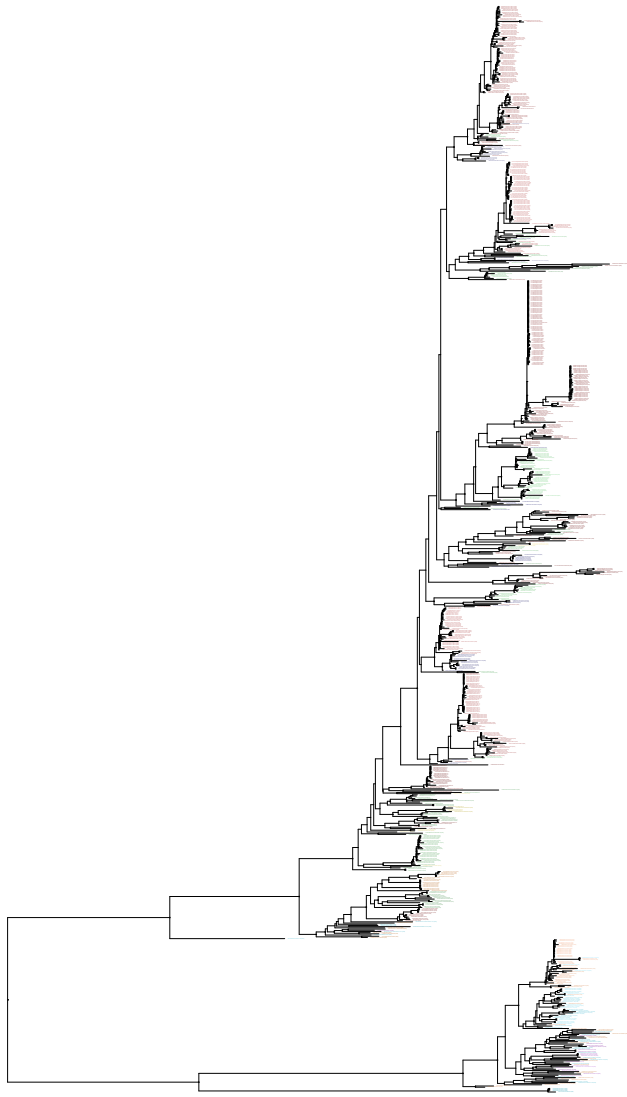

B

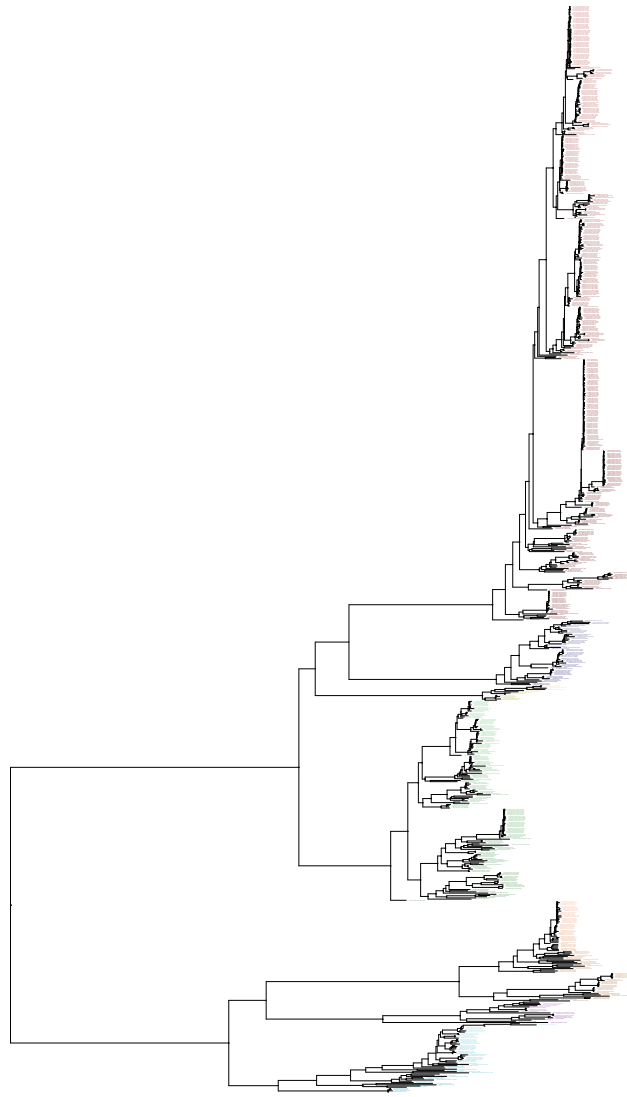

C

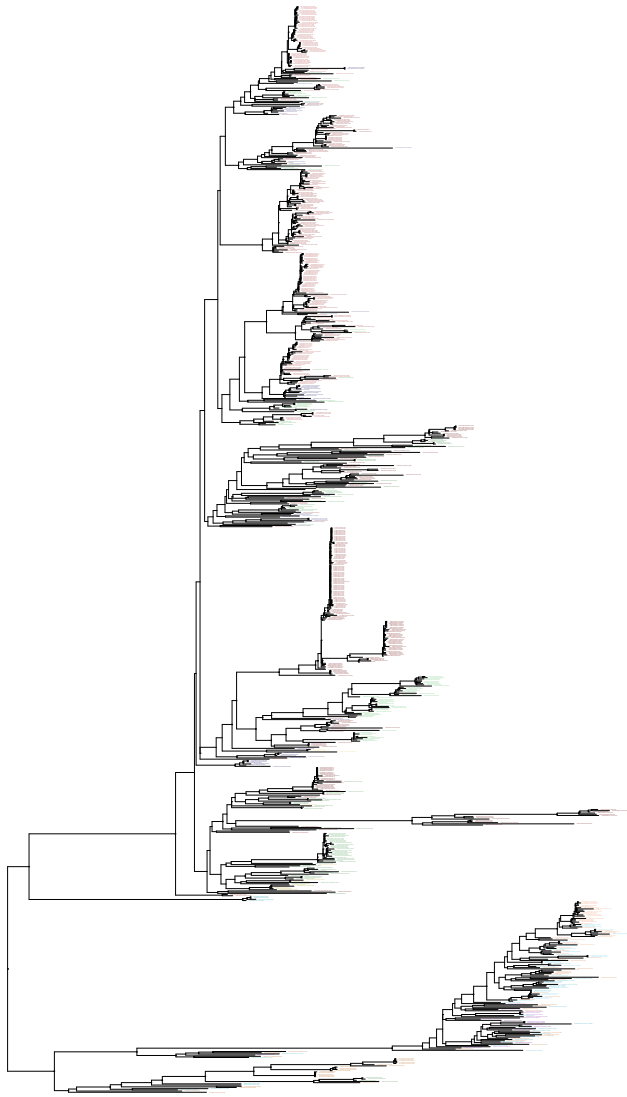

D

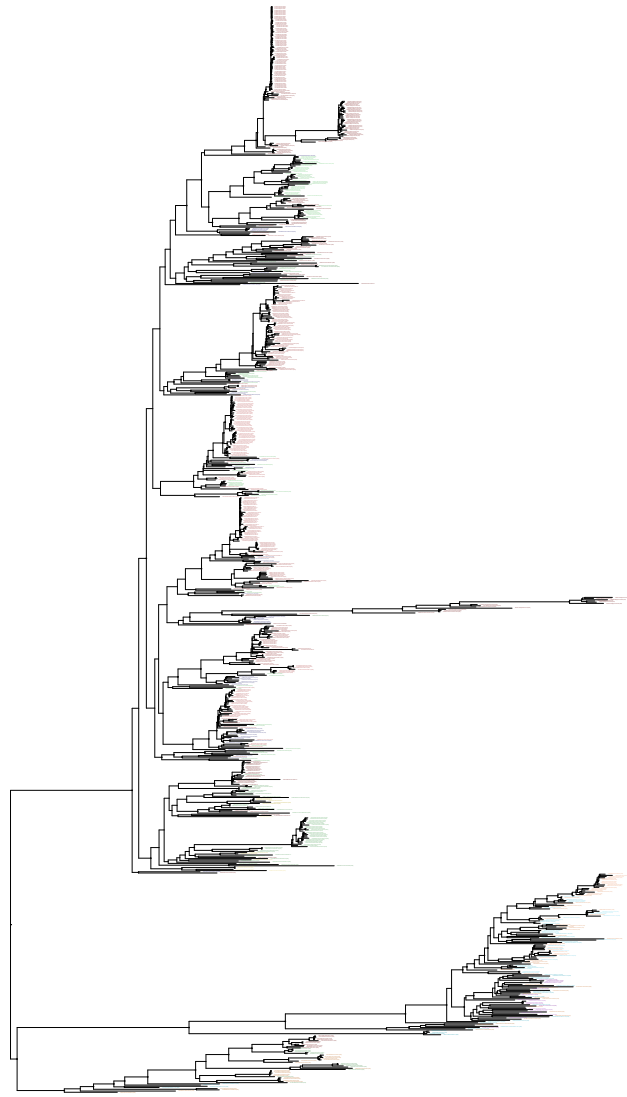

Supplement: Supplementary file 1 [file viruses-18-00262-s001.zip › submission_supplements/FigureS1.pdf]

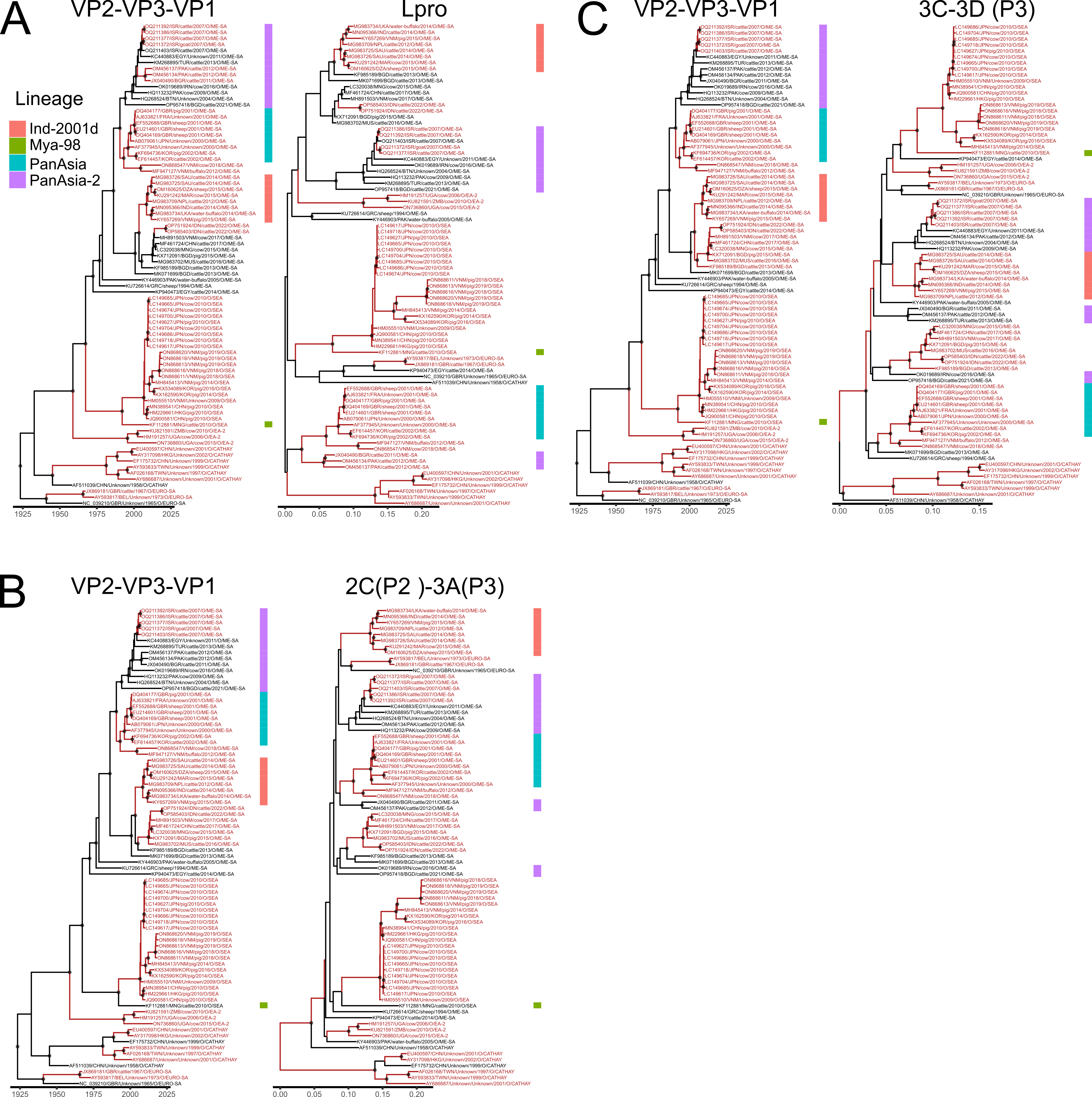

Supplement: Supplementary file 1 [file viruses-18-00262-s001.zip › submission_supplements/FigureS10.png]

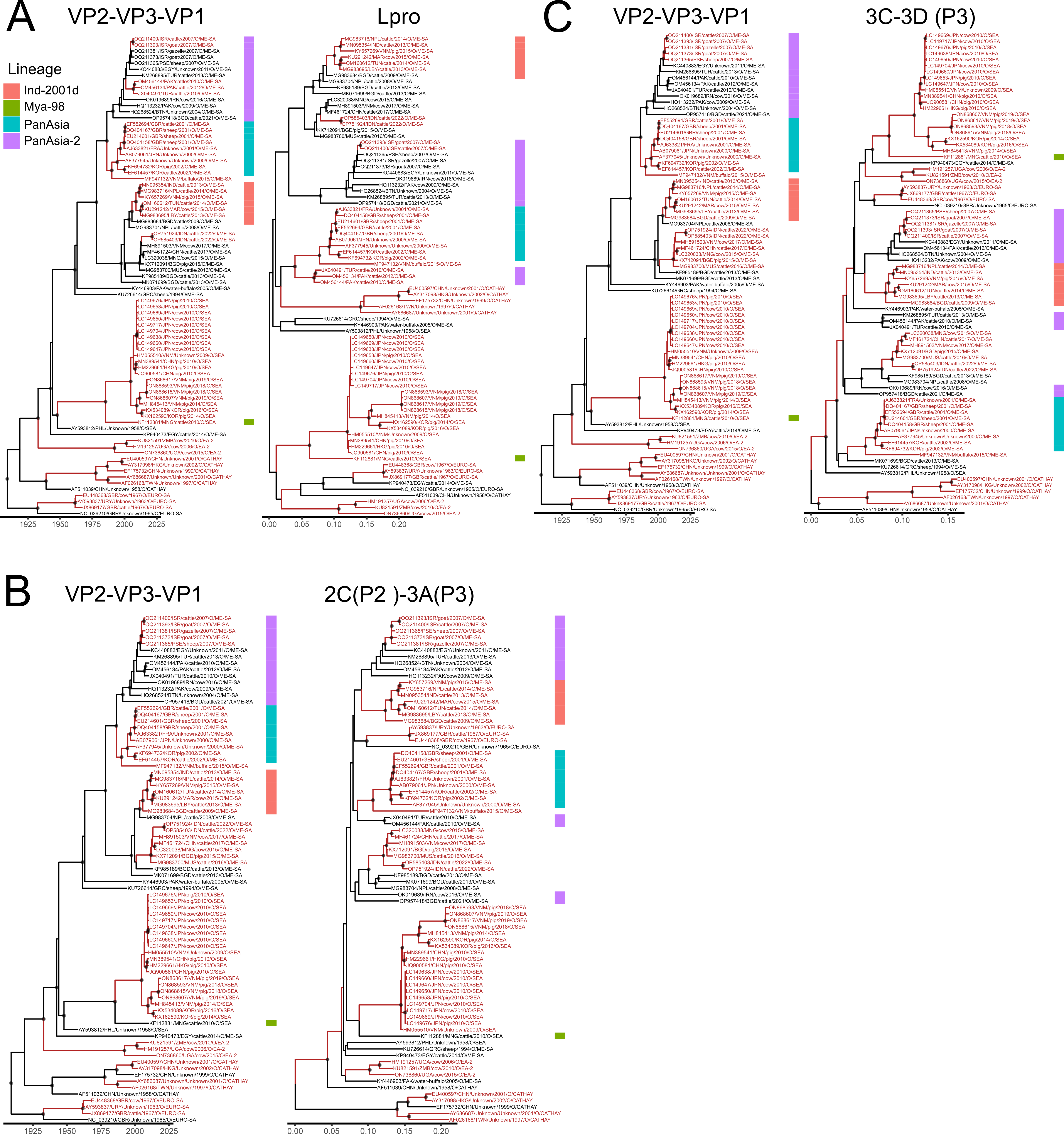

Supplement: Supplementary file 1 [file viruses-18-00262-s001.zip › submission_supplements/FigureS11.png]

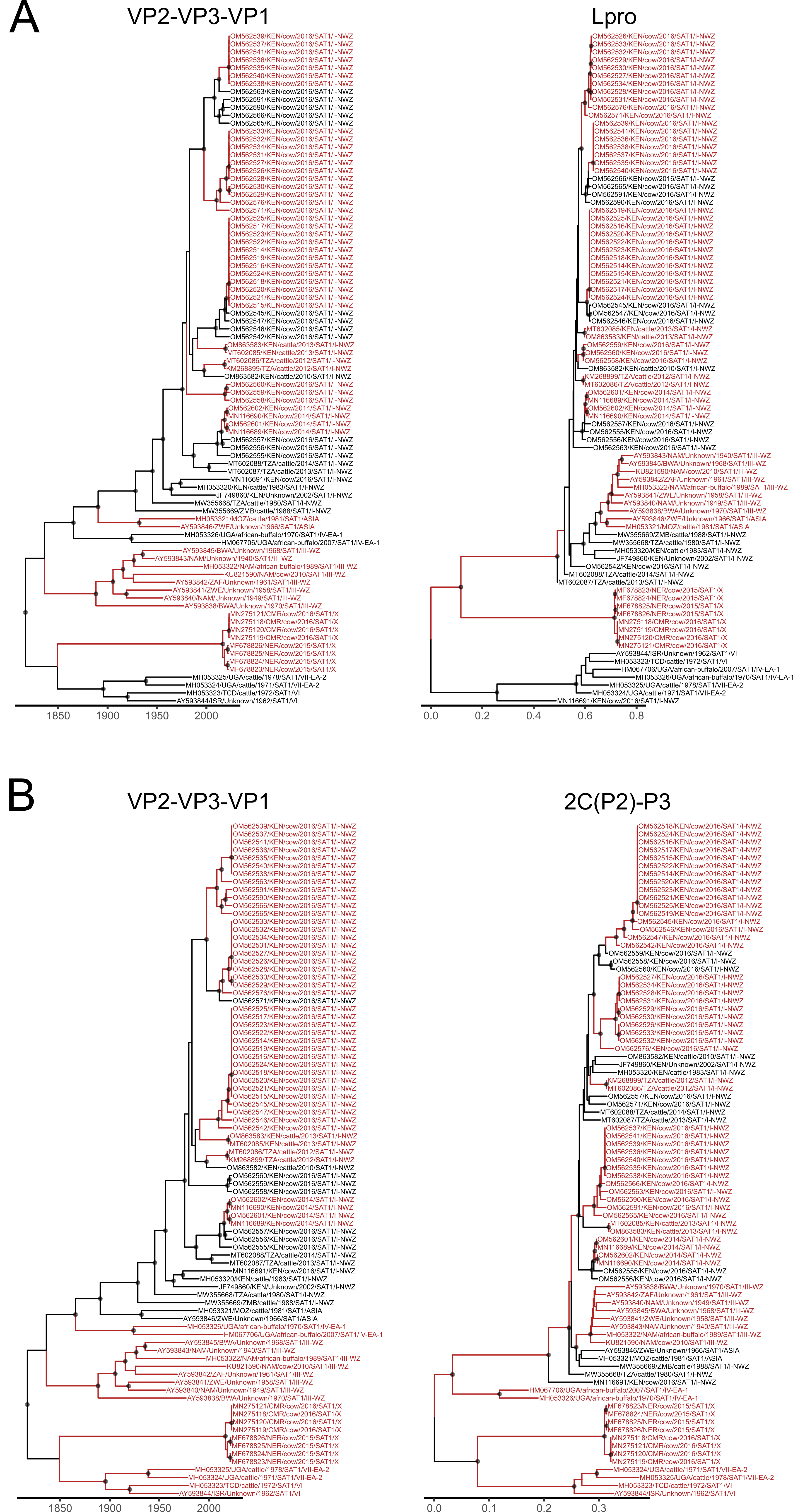

Supplement: Supplementary file 1 [file viruses-18-00262-s001.zip › submission_supplements/FigureS12.png]

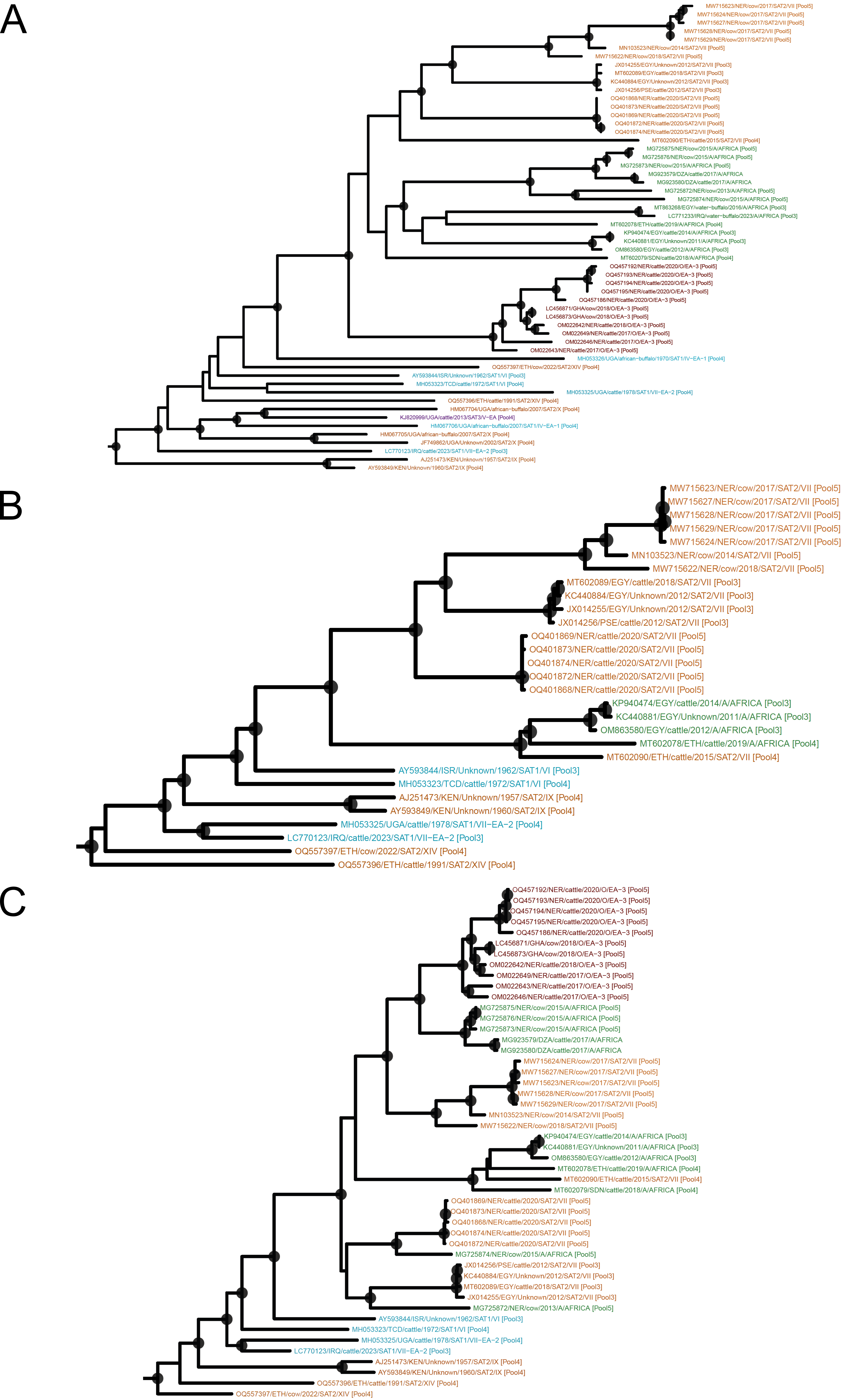

Supplement: Supplementary file 1 [file viruses-18-00262-s001.zip › submission_supplements/FigureS2.png]

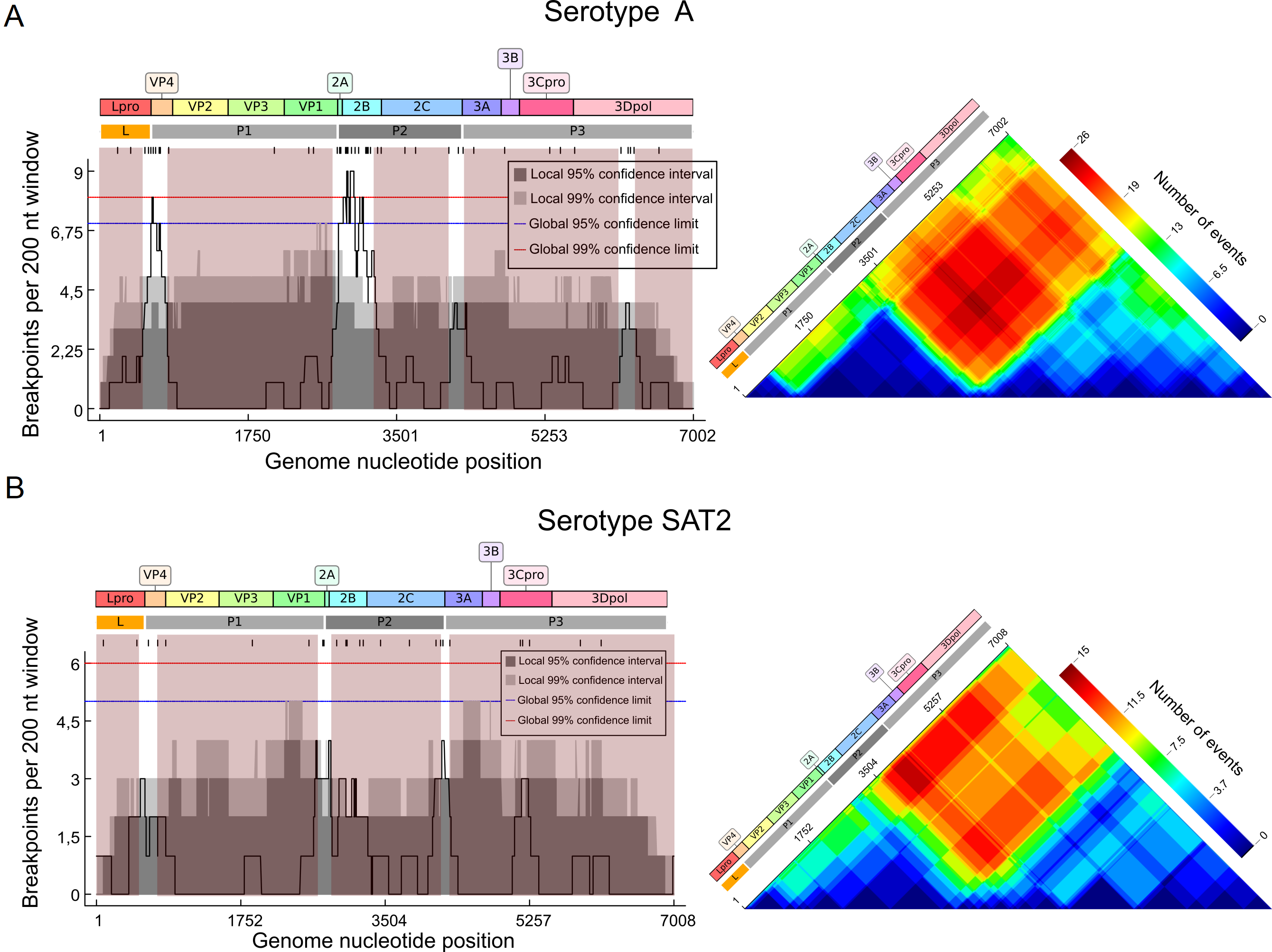

Supplement: Supplementary file 1 [file viruses-18-00262-s001.zip › submission_supplements/FigureS3.png]

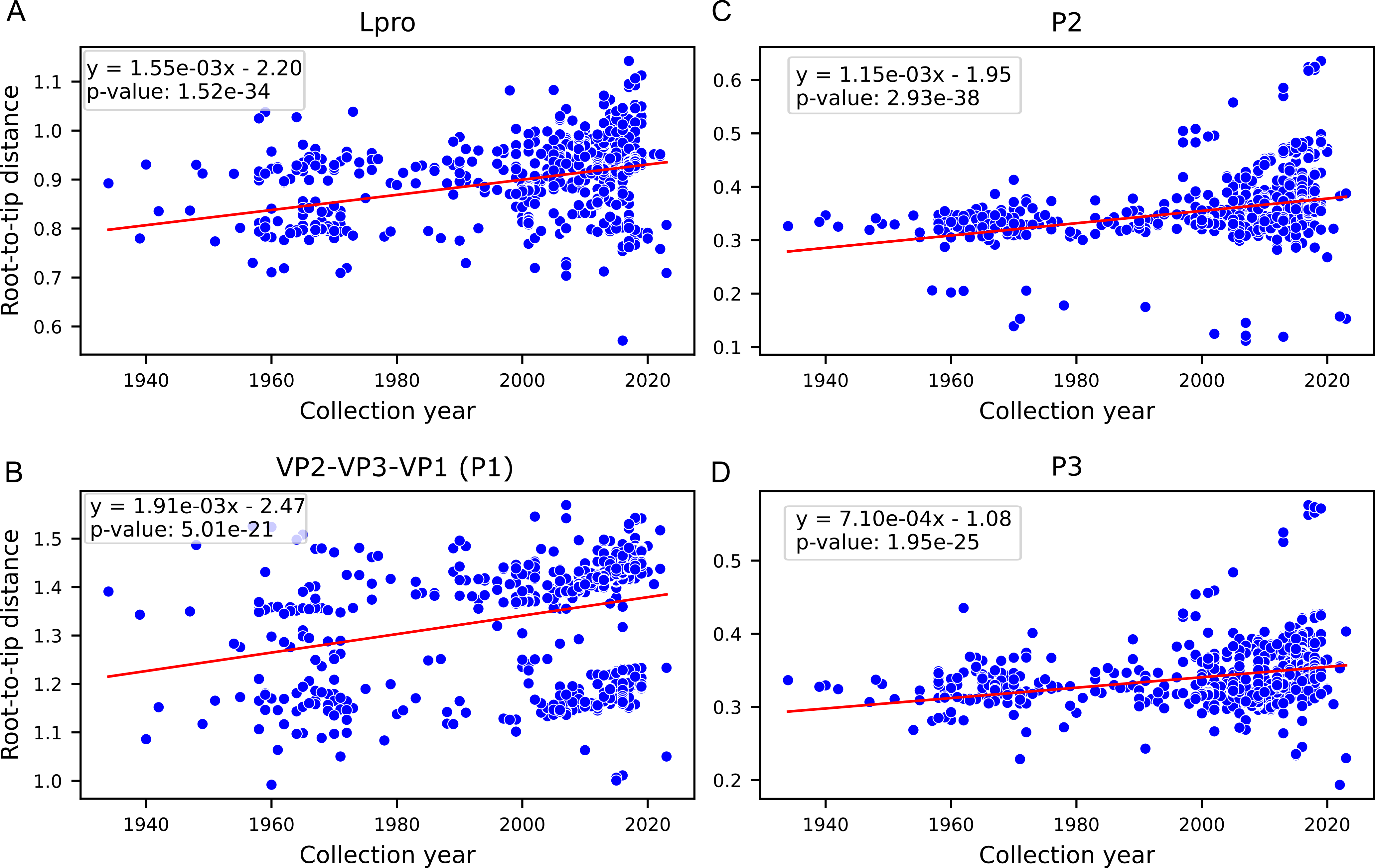

Supplement: Supplementary file 1 [file viruses-18-00262-s001.zip › submission_supplements/FigureS4.png]

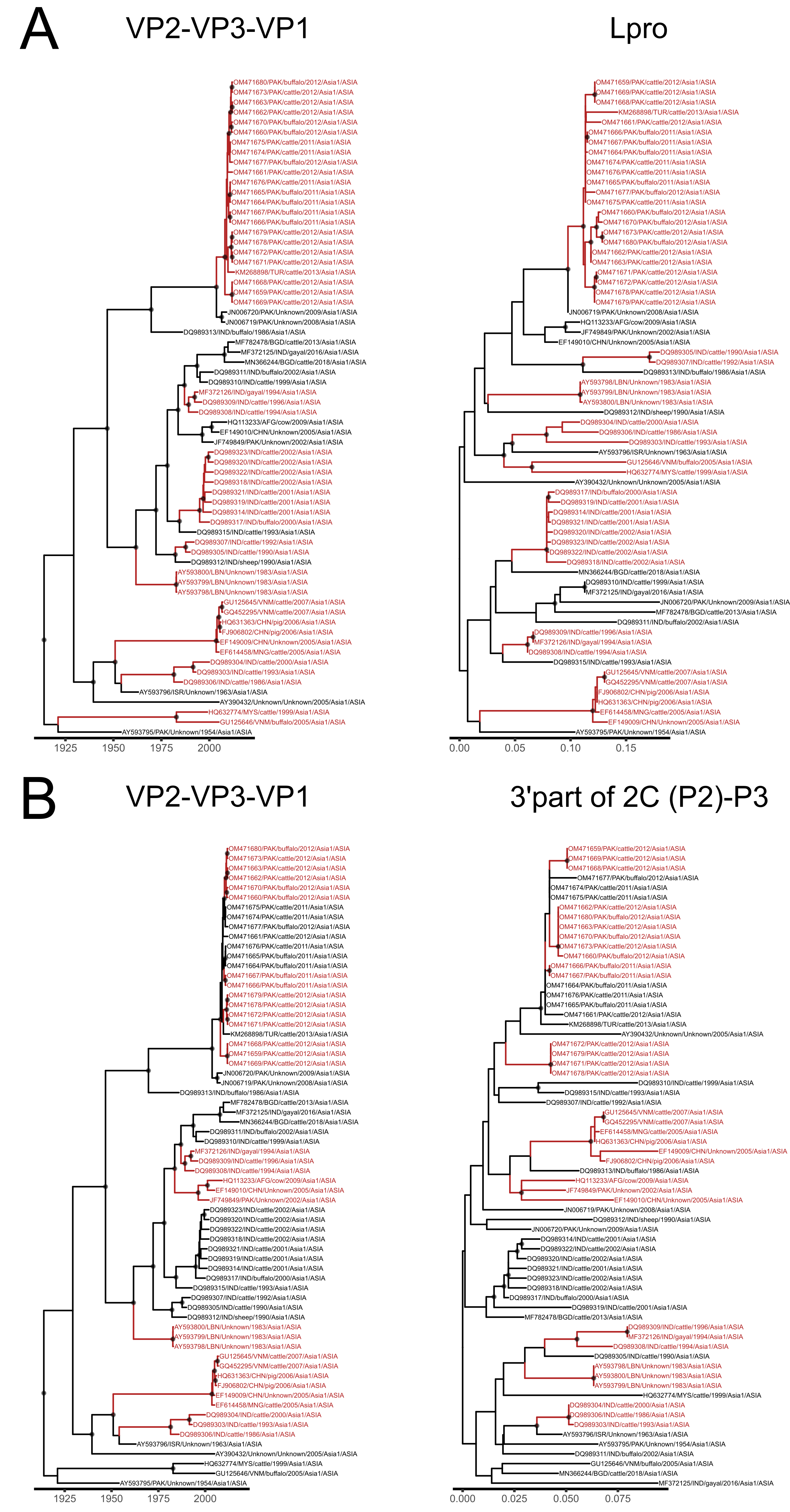

Supplement: Supplementary file 1 [file viruses-18-00262-s001.zip › submission_supplements/FigureS6.png]

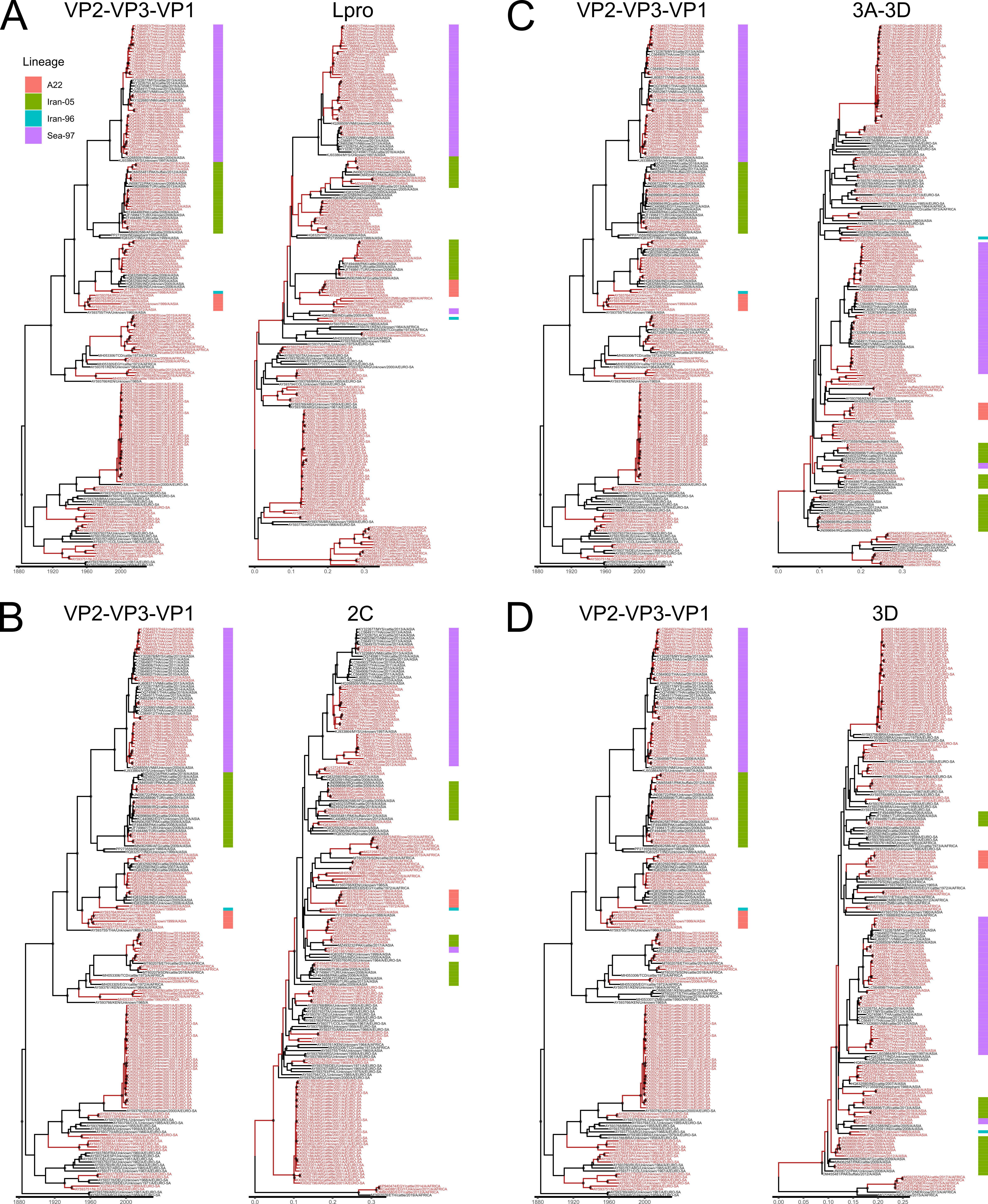

Supplement: Supplementary file 1 [file viruses-18-00262-s001.zip › submission_supplements/FigureS7.png]

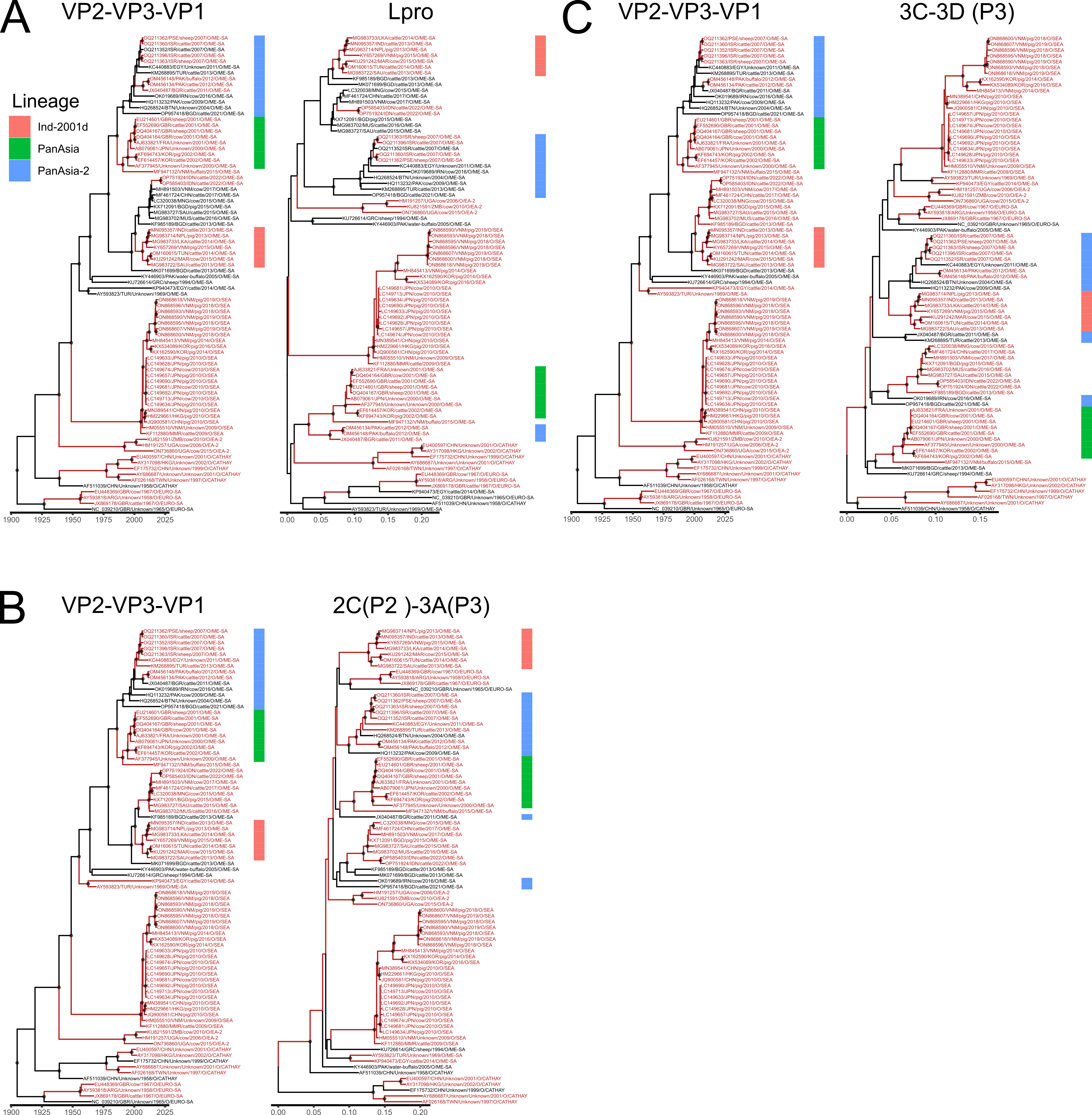

Supplement: Supplementary file 1 [file viruses-18-00262-s001.zip › submission_supplements/FigureS8.png]

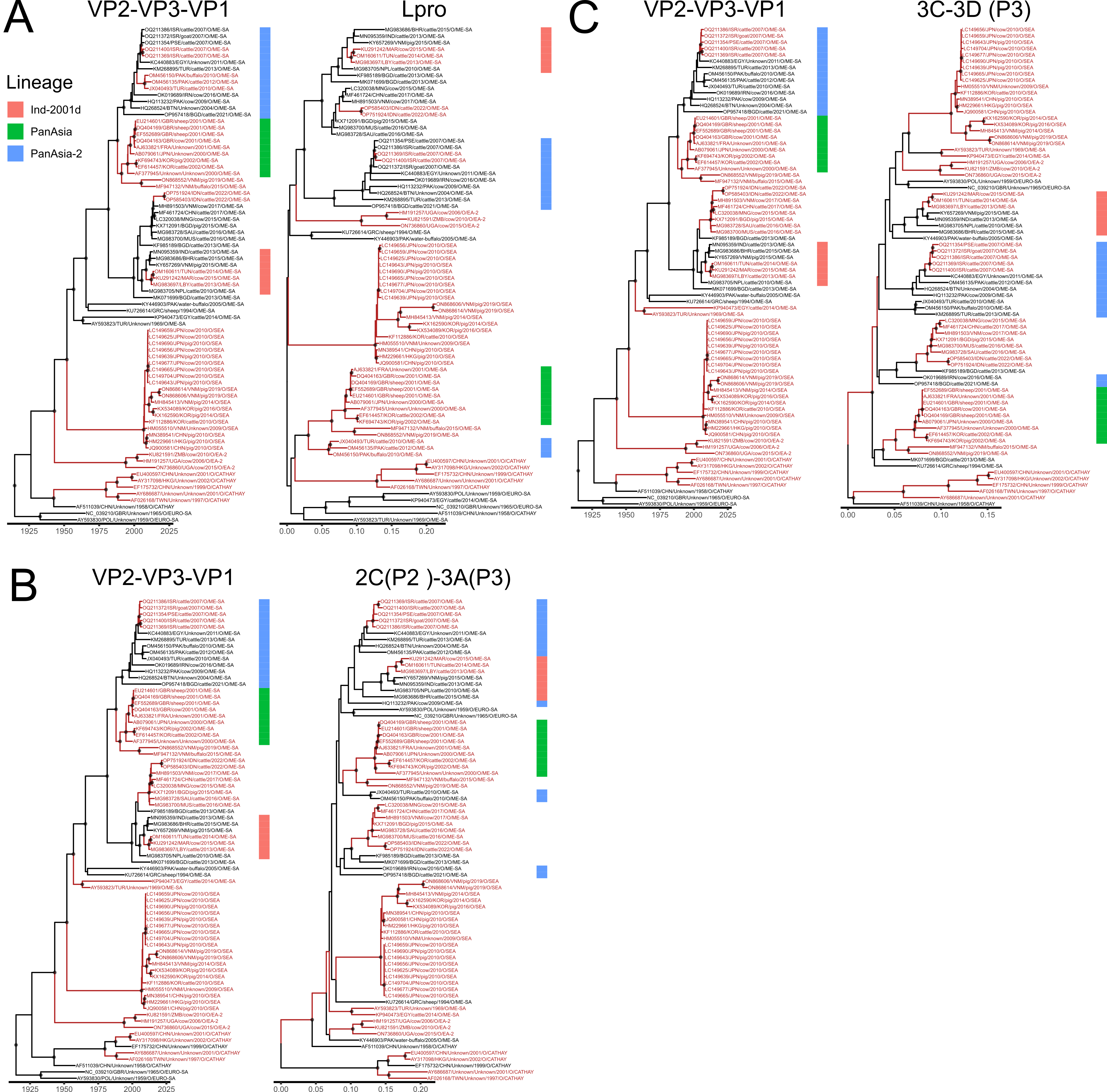

Supplement: Supplementary file 1 [file viruses-18-00262-s001.zip › submission_supplements/FigureS9.png]
